# Inhibitory Neurotransmission Is Sex-Dependently Affected by Tat Expression in Transgenic Mice and Suppressed by the Fatty Acid Amide Hydrolase Enzyme Inhibitor PF3845 via Cannabinoid Type-1 Receptor Mechanisms

**DOI:** 10.3390/cells11050857

**Published:** 2022-03-02

**Authors:** Changqing Xu, Barkha J. Yadav-Samudrala, Callie Xu, Bhupendra Nath, Twisha Mistry, Wei Jiang, Micah J. Niphakis, Benjamin F. Cravatt, Somnath Mukhopadhyay, Aron H. Lichtman, Bogna M. Ignatowska-Jankowska, Sylvia Fitting

**Affiliations:** 1 Department of Psychology & Neuroscience, University of North Carolina at Chapel Hill, Chapel Hill, NC 27599, USA; changqi@email.unc.edu (C.X.); barkhaj@email.unc.edu (B.J.Y.-S.); cxlblue44@gmail.com (C.X.); 2 Department of Chemistry & Biochemistry, North Carolina Central University, Durham, NC 27707, USA; bnath@eagles.nccu.edu (B.N.); tmistry@eagles.nccu.edu (T.M.); smukhopadhyay@nccu.edu (S.M.); 3 Department of Microbiology and Immunology, Medical University of South Carolina, Charleston, SC 29425, USA; jianw@musc.edu; 4 Division of Infectious Diseases, Department of Medicine, Medical University of South Carolina, Charleston, SC 29425, USA; 5 Department of Chemical Physiology, Scripps Research Institute, La Jolla, CA 92037, USA; miip@lundbeck.com (M.J.N.); cravatt@scripps.edu (B.F.C.); 6 Department of Pharmacology & Toxicology, Virginia Commonwealth University, Richmond, VA 23298, USA; aron.lichtman@vcuhealth.org; 7 Neuronal Rhythms in Movement Unit, Okinawa Institute of Science and Technology, Onna 904-0495, Japan; bogna.ignatowska@oist.jp

**Keywords:** endocannabinoids, neuroHIV, fatty acid amide hydrolase, Tat transgenic mice, inhibitory postsynaptic potentials, PF3845, cannabinoid type 1 receptor, anandamide, non-eCB lipids, endocannabinoid enzyme inhibitor

## Abstract

(1) Background. The endocannabinoid (eCB) system, which regulates physiological and cognitive processes, presents a promising therapeutic target for treating HIV-associated neurocognitive disorders (HAND). Here we examine whether upregulating eCB tone has potential protective effects against HIV-1 Tat (a key HIV transactivator of transcription) protein-induced alterations in synaptic activity. (2) Methods. Whole-cell patch-clamp recordings were performed to assess inhibitory GABAergic neurotransmission in prefrontal cortex slices of Tat transgenic male and female mice, in the presence and absence of the fatty acid amide hydrolase (FAAH) enzyme inhibitor PF3845. Western blot and mass spectrometry analyses assessed alterations of cannabinoid receptor and enzyme protein expression as well as endogenous ligands, respectively, to determine the impact of Tat exposure on the eCB system. (3) Results. GABAergic activity was significantly altered upon Tat exposure based on sex, whereas the effectiveness of PF3845 to suppress GABAergic activity in Tat transgenic mice was not altered by Tat or sex and involved CB_1_R-related mechanisms that depended on calcium signaling. Additionally, our data indicated sex-dependent changes for AEA and related non-eCB lipids based on Tat induction. (4) Conclusion. Results highlight sex- and/or Tat-dependent alterations of GABAergic activity and eCB signaling in the prefrontal cortex of Tat transgenic mice and further increase our understanding about the role of FAAH inhibition in neuroHIV.

## 1. Introduction

Despite the successful introduction of combination antiretroviral therapy (cART) [1,2,3], the prevalence of mild to moderate forms of human immunodeficiency virus type 1 (HIV-1)-associated neurocognitive disorders (HAND) remains high [4,5,6,7,8,9]. Symptoms of HAND in the post-cART era are specifically associated with cortical brain structures, such as the prefrontal cortex (PFC) [10,11], and include impairments in executive function, memory consolidation, decision-making, and attention [6,10,11,12,13]. The pathogenesis underlying HAND has been suggested to involve an early viral protein, HIV-1 transactivator of transcription (Tat), which is continually secreted from infected cells in the central nervous system (CNS) despite cART [14]. Various preclinical in vivo and in vitro studies demonstrate Tat’s ability to induce inflammation, excitotoxicity, dendritic damage, and synaptic alteration [15,16,17,18,19,20,21,22], all of which contribute to HAND development and is recapitulated in Tat rodent models [23,24,25,26,27,28,29,30,31,32,33,34,35,36,37,38,39,40,41,42,43]. Studies examining cognitive function associated with PFC tasks in the Tat transgenic mouse model have shown inhibitory control deficits, increased novelty exploration, and pre-attentive filtering deficits [32,43] that coincide with inflammatory responses as well as decreases in inhibitory pre- and post-synaptic proteins [43]. Alterations of the GABAergic system by Tat have been demonstrated in various mouse brain structures [28,30,40,44,45,46] and have further been implicated in HAND pathology in people living with HIV (PWH) [28,30,31,40,44,45,47,48,49]. Nevertheless, little is known about whether sex may differentially contribute to HIV/Tat-induced alterations on inhibitory GABAergic neurotransmission in the PFC and how endocannabinoid tone might be altered in the context of HAND.

As HAND is associated with inflammatory and neurotoxic insults [50,51,52], the endocannabinoid (eCB) system, which regulates both immune function and cognition, has high therapeutic potential for treating the consequences of HIV-1 infection because of the anti-oxidative, anti-excitotoxic, and anti-inflammatory properties of cannabinoids (see reviews, [53,54,55,56]). To avoid site effects induced with cannabinoid receptor agonists due to lack of site specificity and CB_1_R-related psychoactive effects [57], research efforts have specifically focused on the development of drugs targeting components of the endogenous cannabinoid system, including enzymes regulating the biosynthesis and degradation of the two major endogenous cannabinoids N-arachidonoyl ethanolamine (anandamine/AEA) and 2-arachidonoyl glycerol (2-AG) [58,59,60]. Selective enzyme inhibitors of the main AEA-metabolizing enzyme, fatty acid amide hydrolase (FAAH), and of the main 2-AG catabolic enzyme, monoacylglycerol lipase (MAGL), are promising therapeutic tools as they enhance eCB signaling on demand in locations where they are being actively produced, e.g., at the site of injury, to evoke their local neuroprotective effects with minimal off-target effects [61,62,63]. Recent preclinical research studies have demonstrated the protective effects of FAAH and MAGL enzyme inhibitors in a variety of different neurodegenerative disease models [63,64,65], including neuroHIV [19,32,66,67,68,69,70]. Work on the FAAH enzyme inhibitor PF3845 indicated protective effects against Tat-induced synaptodendritic injury and neuronal death in cultured primary frontal cortex neurons in vitro [19,70], as well as against increased neuronal excitability (excitatory postsynaptic currents, EPSCs) in Tat-treated PFC slices ex vivo [32]. Additionally, it is not clear whether HIV-1 proteins, such as Tat, affect the eCB system and its signaling properties. Alterations of the eCB system in neuroHIV have been reported for cannabinoid receptors [32,71,72,73] but less so for endocannabinoid ligands [19,70]. Further, a downregulation of cannabinoid signaling in the presence of neuroHIV has been demonstrated in some preclinical studies [48,74].

Thus, in the present study we were interested in examining Tat effects on inhibitory GABAergic activity and how eCB signaling might be altered in the context of neuroHIV. Whole-cell patch-clamp recordings were conducted to assess inhibitory GABAergic neurotransmission in prefrontal cortex slices of Tat transgenic male and female mice, in the presence and absence of PF3845 bath application. Western blot and mass spectrometry analyses assessed alterations on CB_1_R, FAAH, and MAGL protein expression and eCB and non-eCB lipids, respectively, to determine the impact of HIV-1 Tat exposure on the eCB system.

## 2. Materials and Methods

### 2.1. Animals

Doxycycline (DOX)-inducible, brain-specific HIV-1_IIIB_ Tat_1-86_ transgenic male and female mice (~4–5 months of age) were used in the present study and developed on a C57BL/6J hybrid background described in detail in previous literature [30,75]. All mice received a specially formulated chow containing 6 mg/g DOX (Harlan, Indianapolis, IN, USA, product #: TD.09282), including mice that express the *GFAP-rtTA* and *TRE-tat* genes [Tat(+) mice] to induce Tat expression and control Tat(−) transgenic mice that express only the *GFAP-rtTA* gene and lack the *tat* transgene. Animals were fed the DOX-supplemented food 3 months before experiments were conducted. Mice were group-housed (2–4 mice per cage) on a reversed 12-h light/dark cycle (lights off at 8:00 AM) and had free access to water and chow. The wellbeing of all the animals was monitored by expert veterinarians. Experiments were conducted in accordance with the ethical guidelines defined by the National Institutes of Health (NIH Publication No. 85-23) [76] and all procedures were approved by the University of North Carolina at Chapel Hill (UNC) Institutional Animal Care and Use Committee (IACUC, Protocol ID: 20-108.0, Web ID: 84198).

### 2.2. Slice Electrophysiology Ex Vivo

#### 2.2.1. Prefrontal Cortex (PFC) Slices

Brain PFC slices were prepared from male and female Tat transgenic mice. Brains were removed after decapitation and placed into ice-cold sucrose buffer containing (in mM): 254 sucrose,10 D-glucose, 26 NaHCO_3_, 2 CaCl_2_, 2 MgSO_4_, 3 KCl, and 1.25 NaH_2_PO_4_, saturated with 95% O_2_/5% CO_2_, at pH 7.4, 300 mOsm. Coronal PFC slices (300 μm thick) were cut with a VT 1000S microtome (Leica, Deerfield, IL, USA). Slices were transferred immediately into a holding chamber and were incubated at 32 to 33 °C for a 30-min recovery period in a mixture of 50% sucrose saline and 50% artificial cerebrospinal fluid (aCSF) containing (in mM): 128 NaCl, 10 D-glucose, 26 NaHCO_3_, 2 CaCl_2_, 2 MgSO_4_, 3 KCl, and 1.25 NaH_2_PO_4_. Slices were then placed on a nylon mesh, submerged in normal aCSF bubbled continuously with 95% O_2_/5% CO_2_, and maintained at room temperature (~21–24 °C) until whole-cell patch-clamp recording (30 min to 5 h).

#### 2.2.2. Electrophysiological Recordings

Slices were transferred to a submersion-type recording chamber (Warner Instruments, Hamden, CT, USA) on a Siskiyou 4080P fixed-stage system (Grants Pass, OR, USA), secured beneath a nylon harp, and perfused with aCSF heated to 30 to 33 °C with an inline heater (Warner SC-20, Hamden, CT, USA) at a rate of 2 to 3 mL per min. Recordings were taken from the medial (m)PFC of layer 2/3 as previously shown [45]. Layer 2/3 pyramidal neurons are known to receive dense inhibitory synaptic input from a rich variety of interneurons to provide tight control of neuronal excitability [77,78]. PFC pyramidal neurons were identified visually by using an Axio Examiner A1 microscope (Zeiss, Thornwood, NY, USA) equipped with a 40× water-immersion objective coupled with an infrared differential interference contrast and an integrated Dodt gradient camera system. Whole-cell patch-clamp recordings from mPFC neurons were established using a MultiClamp 700B amplifier (Axon Instruments, Union City, CA, USA). Membrane current and potential signals were digitized and analyzed with Digidata 1550A and pClamp 10.0 systems (Molecular Devices, Sunnyvale, CA, USA). Patch pipettes of ~5 MΩ were pulled with a PC-10 puller (Narishige, Greencale, NY, USA). The pipette solution had the following composition (in mM) unless otherwise stated: 140 KCl, 0.1 CaCl_2_, 5 EGTA, 10 HEPES, 4 ATP-Mg^2+^, 0.4 GTP-2Na^+^, 1 QX314 (Lidocaine N-ethyl bromide), pH 7.2, and 290 mOsm. QX314 was added to the pipette solution to block the GABA_B_R-mediated currents and to prevent the generation of Na^+^-dependent action potentials. Under these conditions, miniature postsynaptic currents (mPSCs) were acquired in aCSF containing tetrodotoxin (TTX, 1 µM) at a holding potential of −70 mV. To record spontaneous inhibitory postsynaptic currents (sIPSCs) and mIPSCs, glutamate receptor antagonists DNQX (20 µM) and AP-5 (20 µM) were added to aCSF. Drugs were administered by bath application. Synaptic currents were collected for 5 min for each experimental condition. Access resistance (<25 MΩ) was regularly monitored during recordings, and cells were rejected if resistance changed >15% during the experiment. If the access resistance increased during course of the experiment and caused significant reductions in the synaptic current amplitudes, efforts were made to improve access (such as applying additional suction or slight positive pressure); if this failed, the experiment was discontinued.

#### 2.2.3. Acquisition and Analysis of Synaptic Currents

Spontaneously occurring synaptic currents were filtered at 2 kHz and digitized at 10 kHz using Digidata 1550A. Off-line analysis of synaptic currents was performed using the Minianalysis software (Version 6.0.8; Synaptosoft, Decatur, GA, USA). Synaptic currents were screened automatically using an amplitude threshold of 3 pA. Events were then visually screened to ensure that the analysis was not distorted by changes in noise level or by membrane fluctuations. If the background noise increased during the recording, data from that cell were discarded.

### 2.3. Treatments and Drugs

Treatments included the FAAH enzyme inhibitor PF3845 (1 μM, Dr. Benjamin Cravatt), the CB_1_R antagonist SR141716A (SR1, 1 μM, Tocris, Ellisville, MO, USA) and the CB_2_R antagonist AM630 (1 μM, Tocris). Cannabinoid concentrations were chosen based on preliminary experiments (data not shown) and previous studies from our laboratory [45]. AP-5 (DL-2-amino-5-phosphonovaleric acid, NMDA receptor antagonist, 20 µM), DNQX (6,7-dinitroquinoxaline-2,3-dione, AMPA and kainate receptor antagonists, 20 µM), and TTX (tetrodotoxin, 1 µM) were purchased from Tocris. All drugs were dissolved in dimethyl sulfoxide (DMSO), except for TTX, and AP-5, which were dissolved in distilled water. All stock solutions were stored at −80 °C as frozen aliquots for less than one month. Drugs were administered by bath application. AP-5, DNQX, and TTX were bath applied 20 min prior to, and for the duration of the experiment. For the experimental manipulation of extracellular calcium, cadmium chloride (CdCl_2_, 200 µM, Sigma-Aldrich, St. Louis, MO, USA) was applied to the bath, which blocks high and low threshold voltage-dependent calcium channels, or aCSF was used without calcium. For the manipulation of intracellular calcium levels, the endoplasmic reticulum calcium pump inhibitor thapsigargin (1 µM, Sigma-Aldrich, St. Louis, MO, USA) was bath applied to the slices that depletes the intracellular calcium stores.

### 2.4. Western Blot Analysis

For the western blot analysis, tissue from the PFC of male and female Tat transgenic mice were freshly harvested and homogenized on ice in an appropriate volume of ice-cold RIPA Lysis and Extraction buffer (G-Bioscience, St. Louis, MO, USA) with protease inhibitor cocktail (VWR Life Science, Cleveland, OH, USA). Homogenized tissue lysates were centrifuged at 10,000× *g* for 10 min at 4 °C. To determine sample protein concentration a BCA protein assay was used. Laemmli buffer (1:5) was used to suspend protein lysates and denatured at 95 °C for 3 min. Using BioRad Protean 3 mini apparatus, equal amounts of protein (10 μg/lane) were resolved in 10% SDS-PAGE at a 150 volt for 1 h. Electrophoretic transfer of proteins from the gel to nitrocellulose membranes was carried out in 10 mM CAPS buffer with 0.01% SDS, pH 11, overnight at 0–4 °C at 20 V using a Bio-Rad Trans-Blot Cell equipped with a cooling coil. Blots were washed with Tris-buffered saline (TBS), incubated with blocking buffer (5% nonfat dry milk plus 5% normal goat serum in TBS) for 1 h at room temperature followed by incubation with affinity-purified anti-CB_1_R (rabbit polyclonal; Chemical, Ann Arbor, MI, USA; #101500; 1:1000 dilution for 3 h), anti-FAAH1 (mouse monoclonal; Abcam, Waltham, MA, USA; ab54615; 1:1000 dilution for 3 h), or anti-MAGL (rabbit polyclonal; Abcam, ab24701; 1:1000 dilution for 3 h) at room temperature, washed 2× with TBS, and then incubation with anti β-Actin (anti-mouse or anti-rabbit from Cell Signaling Technology, Danvers, MA; #3700 or #4970; 1:1000) for 3 h, washed 3× with TBS (TBS with 0.1% Tween 20), and incubated with horseradish peroxidase-coupled anti-rabbit and/or anti-mouse IgG sequentially (with 1× TBS wash in between) for 1 h at room temperature. Immunoreactive bands were detected by ECL reaction (Amersham, Buckinghamshire, UK) from the blot using BioRad Gel Doc XR+ system and image acquiring software (Image Lab ver. 5.1, Hercules, CA, USA). Densitometric analysis was carried out using a modified version (version 1.59) of the Scion Image software (Scion Corporation, Frederick, MD, USA).

### 2.5. Analysis of Endocannabinoids (eCBs) and Other Lipids

Endogenous cannabinoid ligands, including the two main eCBs AEA and 2-AG as well as nine related lipids, including *N*-arachidonoyl glycine (NAGly), peroxisome proliferator activator receptor (PPAR) ligands, such as *N*-oleoyl ethanolamide (OEA) and *N*-palmitoyl ethanolamide (PEA), and 2-linoleoyl glycerol (2-LG), were quantified via mass spectrometry in the PFC of male and female Tat transgenic mice. Immediately following cervical dislocation and decapitation, brains were removed and the PFC was rapidly dissected, frozen in liquid nitrogen, and stored at −80 °C until use, as described previously [79]. PFC samples from the right hemisphere were processed, and substrates were quantified in a similar manner to previous studies [80]. Briefly, frozen PFC samples stored in microfuge tubes were removed from the −80 °C freezer and immediately placed on dry ice. Each entire sample was then weighed in a TissueLyzer tube with stainless steel ball (Qiagen, Hilden, Germany), the weight was recorded, and the samples were placed on dry ice until homogenization. Immediately before homogenization, 440 μL of ice-cold methanol (Fisher Scientific, Fair Lawn, NJ, USA), 50 μL of internal standard containing 50 ng/mL of 2-AG-d5, 5 ng/mL of AEA-d4, 5 ng/mL of OEA-d4 (Cayman Chemical, Ann Arbor, MI, USA), and 10 μL of 5 mg/mL of BHT (Sigma-Aldrich, St. Louis, MO, USA) in methanol antioxidant solution was added. Samples were then homogenized at 50 Hz for 2 min (Qiagen TissueLyzer LT, Germantown, MD, USA). Immediately after homogenization, samples were placed in a microcentrifuge at 14,000 RPM for 10 min at 4 °C. The supernatant was removed and placed in an MRQ reduced surface activity vial (Microsolv, Leland, NC, USA) and analyzed immediately by liquid chromatography coupled with tandem mass spectrometry (LC/MS/MS; ref. [80]).

### 2.6. Statistical Analysis

Data are presented as raw data or percent change from control (mean ± SEM) and tested for normality with Shapiro–Wilk test. Data sets from western blot analysis and mass spectrometry were analyzed by a two-way analysis of variance (ANOVA) with sex (2 levels: male, female) and genotype (2 levels: Tat(−) mice, Tat(+) mice) as between-subject factors. ANOVAs were followed by planned comparisons or Bonferroni post hoc correction for multiple comparisons as necessary (SPSS Statistics 25; IBM, Chicago, IL, USA; and/or GraphPad 8.0, San Diego, CA, USA). For electrophysiology data, in addition to two-way ANOVAs, data were analyzed by three-way repeated measures ANOVAs when appropriate, including sex and genotype as between-subject factors and drug bath exposure (i.e., PF3845; SR141716A, AM630, 0 Ca^2+^, CdCl_2_, Thapsigargin) as a within-subject factor. ANOVAs were followed by planned comparisons or Bonferroni post hoc correction for multiple comparisons as necessary. Electrophysiology data that are presented as percent change from control (% of control) were further analyzed using a one-sample *t*-test (control, 0%) to assess significance from baseline. To correct for multiple comparisons Bonferroni correction was used. Sample size is indicated for all electrophysiology experiments as (cells/mice). An alpha level of *p* < 0.05 was considered significant for all statistical tests.

## 3. Results

### 3.1. Inhibitory GABAergic Neurotransmission Is Altered in Tat Transgenic mPFC Brain Slices in a Sex-Dependent Manner

**Figure 1:**
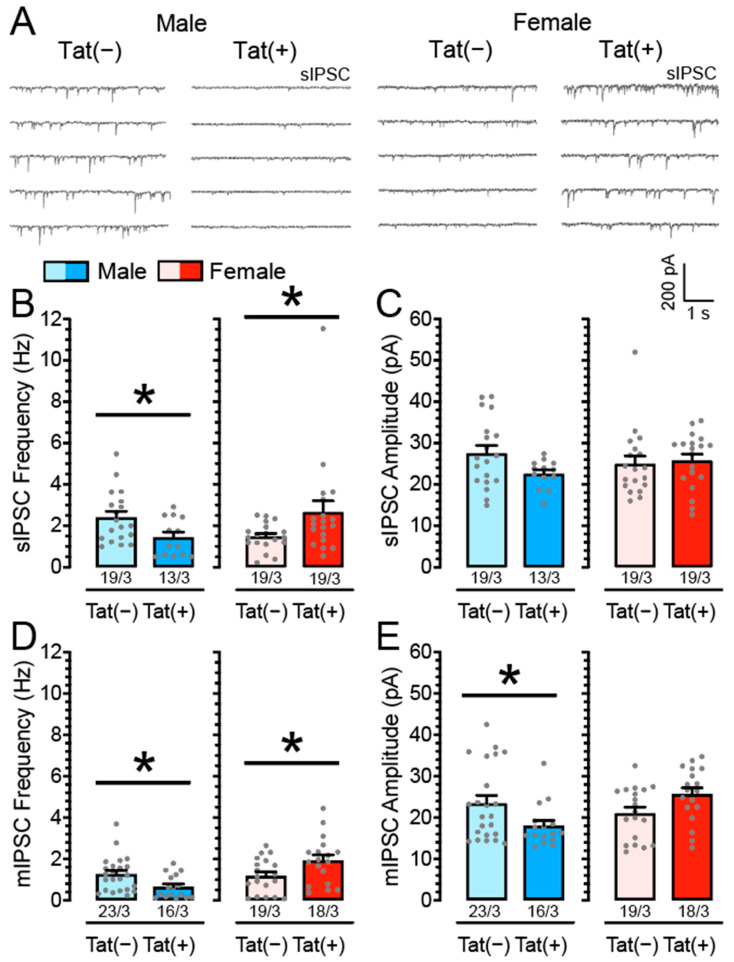
Tat induction alters the frequency and partially the amplitude of IPSCs in mPFC neurons in a sex-dependent manner. (**A**) Representative traces of sIPSCs in female and male Tat transgenic mPFC brain slices. (**B**) Tat induction significantly decreased mean sIPSC frequency for Tat(+) males compared to Tat(−) males, whereas a significant increase in sIPSC frequency was demonstrated for Tat(+) females compared to their respective Tat(−) counterparts. (**C**) No significant effects were noted on the mean amplitude of sIPSCs. (**D**) Similarly, for mIPSCs, Tat(+) males showed decreased mIPSC frequency compared to Tat(−) males and Tat(+) females showed increased mIPSC frequency compared to Tat(−) females. (**E**) For the mean amplitude of mIPSCs only Tat(+) males demonstrated decreased mIPSC amplitude compared to Tat(−) males. Female Tat transgenic mice did not significantly differ from each other. Data are raw data (mean ± SEM) separated by sex and genotype. Statistical significance was assessed by ANOVA followed by planned comparisons; * *p* < 0.05. In all panels, sample size is indicated as (cells/mice).

To explore the effects of Tat induction on spontaneous and miniature GABA_A_ receptor-mediated inhibitory postsynaptic currents (sIPSCs and mIPSCs, respectively), we performed patch-clamp recordings on mPFC pyramidal neurons from Tat transgenic mice (*n* = 13–23 neurons, 3 mice per group and sex) in the presence of DNQX and AP-5 (Figure 1).

Two-way ANOVAs were conducted with sex (2 levels: male, female) and genotype (2 levels: Tat(−), Tat(+)) as between-subject factors. For sIPSC frequency, no significant main effects were noted but a significant sex x genotype interaction, *F*(1, 66) = 8.61, *p* = 0.005 (Figure 1B). Planned comparisons demonstrated decreased sIPSC frequency for Tat(+) males (*M* = 1.44, *SEM* = 0.25, *n* = 13/3) compared to Tat(−) males (*M* = 2.41, *SEM* = 0.29, *n* = 19/3; *p* = 0.025), whereas Tat(+) females showed increased sIPSC frequency (*M* = 2.66, *SEM* = 0.55, *n* = 19/3) compared to Tat(−) females (*M* = 1.48, *SEM* = 0.15, *n* = 19/3; *p* = 0.046). No significant main effects or interaction were noted for sIPSC amplitude (all *p*’s > 0.05; Figure 1C).

To assess mIPSCs, TTX was added to the bath to eliminate large-amplitude, action potential-dependent IPSCs. A two-way ANOVA revealed a significant main effect for sex, *F*(1, 72) = 8.76, *p* = 0.004, with females demonstrating increased mIPSC frequency (*M* = 1.55, *SEM* = 0.17, *n* = 37/6) compared to males (*M* = 1.01, *SEM* = 0.13, *n* = 39/6). Further, a significant sex x genotype interaction was noted on mIPSC frequency, *F*(1, 72) = 11.23, *p* = 0.001, with Tat(+) males indicating decreased mIPSC frequency (*M* = 0.65, *SEM* = 0.14, *n* = 16/3) compared to Tat(−) males (*M* = 1.26, *SEM* = 0.18, *n* = 23/3; *p* = 0.016), whereas Tat(+) females showed increased mIPSC frequency (*M* = 1.93, *SEM* = 0.27, *n* = 18/3) compared to Tat(−) females (*M* = 1.19, *SEM* = 0.19, *n* = 19/3; *p* = 0.029; Figure 1D). For mIPSC amplitude, no significant main effects were noted apart from a significant sex x genotype interaction, *F*(1, 72) = 9.19, *p* = 0.003 (Figure 1E). Planned comparisons demonstrated decreased mIPSC amplitude for Tat(+) males (*M* = 18.01, *SEM* = 1.29, *n* = 16/3) compared to Tat(−) males (*M* = 23.43, *SEM* = 1.89, *n* = 23/3; *p* = 0.038), whereas females did not significantly differ from each other. Thus, whereas for male transgenic mice Tat induction inhibited GABA release (action potential-dependent and action potential-independent), Tat induction increased GABAergic synaptic activity for female mice, predominantly via presynaptic mechanisms.

### 3.2. PF3845 Decreases Inhibitory GABAergic Neurotransmission Independent of Tat Induction and Sex

**Figure 2:**
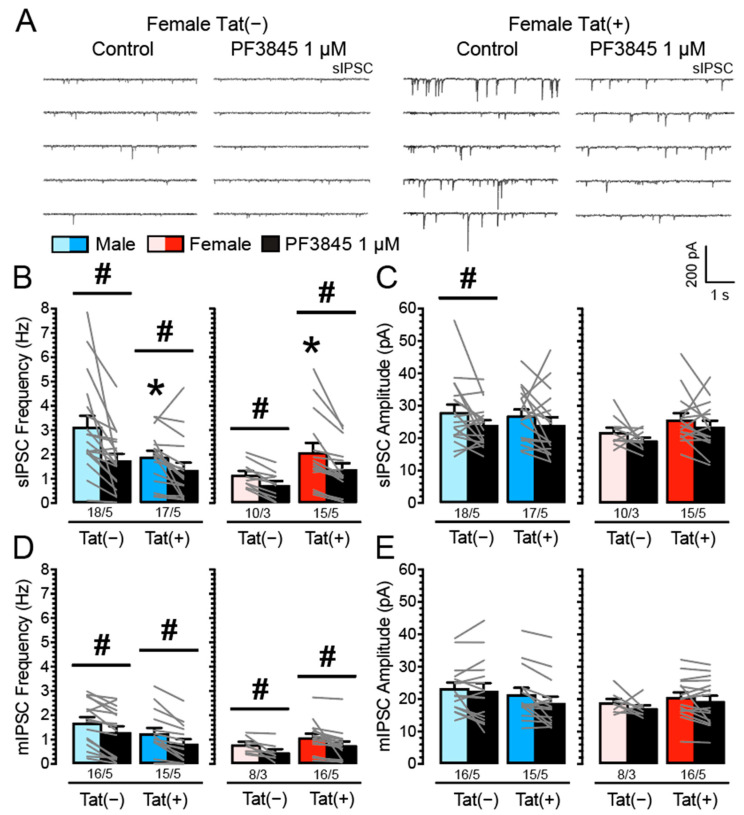
PF3845 decreases the frequency and partially the amplitude of IPSCs in mPFC neurons independent of Tat induction and sex. (**A**) Representative traces show sIPSCs in female Tat(−) and Tat(+) mPFC brain slices before and after PF3845 (1 µM) application. (**B**) Tat(+) males showed decreased sIPSC frequency compared to Tat(−) males, whereas Tat(+) females demonstrated increased sIPSC frequency compared to Tat(−) females. Further, PF3845 significantly decreased the mean sIPSC frequency in Tat(−) and Tat(+) brain slices for each sex with the downregulation being similar across all groups. (**C**) For the mean amplitude of sIPSCs, a significant downregulating effect by PF3845 was noted specifically for Tat(−) males. (**D**) For the mean mIPSC frequency, PF3845 significantly decreased mIPSC frequency in all groups with similar inhibitory effects across. (**E**) For the mean mIPSC amplitude, no significant effects were noted. Data are raw data (mean ± SEM) separated by sex and genotype. Statistical significance was assessed by ANOVA followed by Bonferroni’s post hoc test; * *p* < 0.05 vs. corresponding Tat(−) counterpart; ^#^ *p* < 0.05. In all panels, sample size is indicated as (cells/mice).

It is known that eCBs, such as AEA [81] or 2-AG [82], decrease GABAergic neurotransmission in different brain regions [83]; however, it is unclear if the downregulation of GABA release is altered in the presence of Tat. Thus, we examined the effects of FAAH enzyme inhibition on IPSCs by performing patch-clamp recordings in Tat transgenic mPFC brain slices with bath application of PF3845 (1 µM; *n* = 8–18 neurons, 3–5 mice per group and sex; Figure 2). Three-way mixed ANOVAs were conducted with drug (2 levels: control, PF3845 1 µM) as a within-subject factor and sex and genotype as between-subject factors. For sIPSC frequency, a significant main effect for drug was noted, *F*(1, 56) = 30.55, *p* < 0.001, with an overall inhibition of sIPSC frequency by PF3845 (*M* = 1.37, *SEM* = 0.15; *n* = 60/18) compared to control condition in the absence of PF3845 (*M* = 2.19, *SEM* = 0.20; *n* = 60/18; Figure 2B). 

Further, a significant effect for sex was noted, *F*(1, 56) = 4.99, *p* = 0.029, with males showing higher IPSC frequency (*M* = 2.04, *SEM* = 0.22, *n* = 35/10) compared to females (*M* = 1.42, *SEM* = 0.22, *n* = 25/8), as well as a significant sex x genotype interaction, *F*(1, 56) = 6.56, *p* = 0.013. The sex x genotype interaction indicates decreased sIPSC frequency for Tat(+) males (*M* = 1.63, *SEM* = 0.27, *n* = 17/5) compared to Tat(−) males (*M* = 2.44, *SEM* = 0.33, *n* = 18/5), whereas Tat induction in females indicated increased sIPSC frequency for Tat(+) females (*M* = 1.73, *SEM* = 0.32, *n* = 15/5) compared to Tat(−) females (*M* = 0.94, *SEM* = 0.16, *n* = 10/3; Figure 2B). Lastly, the significant drug x sex x genotype interaction on sIPSC frequency, *F*(1, 56) = 4.08, *p* = 0.048, was further explored by conducting additional analyses on PF3845-induced change (Δ), which, however, revealed no significant differences for sex and/or genotype (Supplemental Figure S1). For sIPSC amplitude, a three-way mixed ANOVA demonstrated a significant effect for drug, *F*(1, 56) = 7.78, *p* = 0.007, with PF3845 treatment inhibiting sIPSC amplitude (*M* = 23.10, *SEM* = 0.97; *n* = 60/18) compared to control condition in the absence of PF3845 (*M* = 26.09, *SEM* = 1.08; *n* = 60/18; Figure 2C).

To assess action potential-independent IPSCs (mIPSCs) TTX was added to the bath to eliminate large-amplitude IPSCs. A mixed three-way ANOVA on mIPSC frequency demonstrated a significant effect for drug, *F*(1, 51) = 48.89, *p* < 0.001, with an overall inhibition of mIPSC frequency by PF3845 (*M* = 0.89, *SEM* = 0.11; *n* = 55/18) compared to control condition in the absence of PF3845 (*M* = 1.26, *SEM* = 0.11; *n* = 55/18; Figure 2D). Further a significant effect for sex was noted, *F*(1, 51) = 5.10, *p* = 0.028, with males indicating higher IPSC frequency (*M* = 1.30, *SEM* = 0.17, *n* = 30/10) compared to females (*M* = 0.81, *SEM* = 0.11, *n* = 25/8; Figure 2D). No other effects and/or interactions were significant. For mIPSC amplitude, a three-way mixed ANOVA demonstrated a significant effect for drug, *F*(1, 51) = 8.06, *p* = 0.006, with PF3845 treatment inhibiting mIPSC amplitude (*M* = 19.79, *SEM* = 1.04; *n* = 55/18) compared to control in the absence of PF3845 (*M* = 21.34, *SEM* = 0.91; *n* = 55/18; Figure 2E). No other effects and/or interactions were significant. Overall, these findings indicate that PF3845 decreases GABAergic neurotransmission presynaptically (sIPSC and mIPSCs frequencies) independent of Tat induction or sex.

### 3.3. PF3845′s Inhibitory Effects on GABAergic Neurotransmission Are Mediated by CB_1_Rs but Not CB_2_Rs

**Figure 3:**
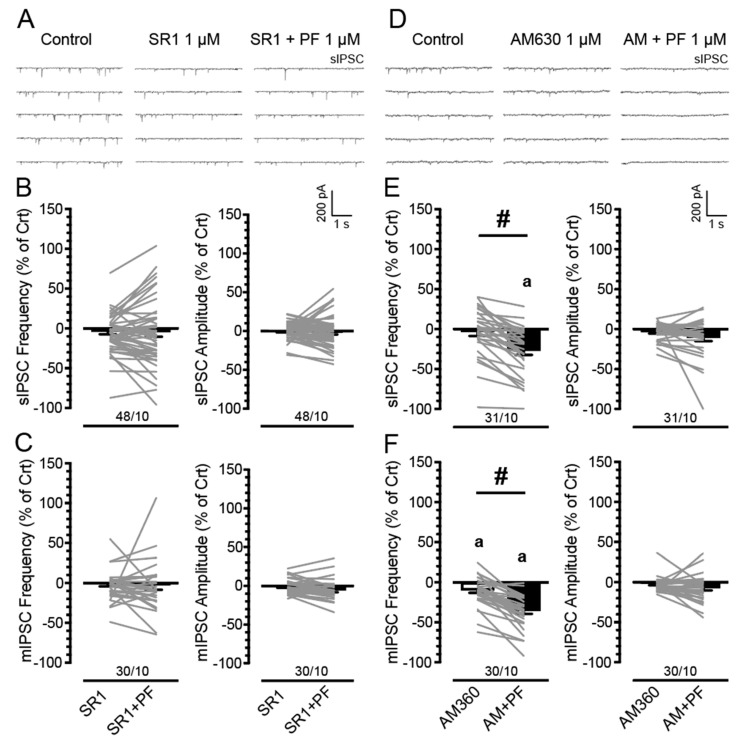
PF3845 effects on the frequency and amplitude of IPSCs (% of control) in mPFC neurons are blocked by CB_1_R antagonist SR141716A but not CB_2_R antagonist AM630. No significant effects were noted for sex and genotype on any sIPSC measure (% of control) and are therefore collapsed across. (**A**) Representative traces show sIPSCs before and after SR141716A (1 µM) ± PF3845 (1 µM) application. (**B**) No significant differences on sIPSC frequency and amplitude (% of control) were noted between the SR141716A alone and SR141716A in combination with PF3845 conditions, indicating the CB_1_R antagonist SR141716A was able to block the downregulating effects of PF3845 on sIPSCs. Further, both conditions were not significantly different from control condition (0%). (**C**) Similarly, no significant effects were noted on the mIPSC frequency and amplitude (% of control). (**D**) Representative traces show sIPSCs before and after AM630 (1 µM) ± PF3845 (1 µM) application. (**E**) There was a significant difference between AM630 alone and AM630 in combination with PF3845 conditions on sIPSC frequency (% of control), indicating pretreatment of AM630 did not prevent the PF3845-induced decreases in the mean frequency of sIPSCs (% of control). This is supported by a significant downregulation of sIPSC frequency for the AM630 in combination with PF3845 condition compared to control (0%), which was not seen for the AM360 alone condition. No significant effects were noted on sIPSC amplitude (% of control). (**F**) Similarly, pretreatment of AM630 did not block the downregulating effects of PF3845 on mIPSC frequency and both conditions significantly decreased mean mIPSC frequency compared to control (0%). No significant effects were noted on mIPSC amplitude (% of control). Data are percent of control data (mean ± SEM) collapsed across sex and genotype. Statistical significance was assessed by ANOVA followed by Bonferroni’s post hoc test; ^#^ *p* < 0.05. One-sample *t*-tests with Bonferroni correction assessed significant changes from control condition (0%), ^a^ *p* < 0.05 vs. control (before treatment, 0%). In all panels, sample size is indicated as (cells/mice); please see Supplemental Table S1 for specific information on sample size for sex and genotype. SR1, SR141716A; PF, PF3845; AM, AM630.

We showed that PF3845 bath application decreased GABAergic neurotransmission independent of Tat induction and sex, but the underlying CBR-related mechanisms were not investigated. Thus, we examined the FAAH enzyme inhibitor PF3845 on IPSCs in Tat transgenic mPFC brain slices with bath application of the CB_1_R antagonist SR141716A (1 µM; *n* = 6–14 neurons, 2–3 mice per group and sex; Figure 3A–C) and the CB_2_R antagonist AM630 (1 µM; *n* = 7–8 neurons, 2–3 mice per group and sex; Figure 3D–F). Three-way mixed ANOVAs were conducted on IPSCs (% of control) with drug (2 levels: CBR antagonist, CBR antagonist + PF3845) as a within-subject factor and sex and genotype as between-subject factors (see Supplemental Result and Figure S2 for analysis and presentation of raw data). As no significant effects and/or interactions were noted for sex and/or genotype on any of the IPSC measures, data are presented combined for sex and genotype (Figure 3).

*Blocking CB_1_Rs withSR141716A (1 µM) bath application.* Three-way mixed ANOVAs on sIPSC (frequency and amplitude; % of control) and mIPSC (frequency and amplitude; % of control) demonstrated no significant effects and/or interactions (Figure 3B,C). Additionally, no significant effects were noted for one-sample *t*-tests (with Bonferroni correction) that assessed significance for percent change from the control condition (0%). These data indicate that the CB_1_R antagonist SR141716A (1 µM) by itself had no significant effect on action potential-dependent and action potential-independent GABA release, and further that SR141716A was able to block the downregulating effects of PF3845 on GABAergic neurotransmission, suggesting PF3845′s inhibitory effects involve CB_1_R-mediated mechanisms.

*Blocking CB_2_Rs withAM630 (1 µM) bath application.* A three-way mixed ANOVA on sIPSC frequency (% of control) demonstrated a significant effect for drug, *F*(1, 27) = 43.09, *p* < 0.001, with AM630 ± PF3845 treatment (*M* = −27.01, *SEM* = 5.42; *n* = 31/10) significantly downregulating sIPSC frequency (% of control) compared to AM630 condition (*M* = −3.41, *SEM* = 5.35; *n* = 31/10; Figure 3E), indicating AM630 pretreatment was not able to block the downregulating effect of PF3845 on sIPSC frequency. No other effects and/or interactions were significant. Further, one-sample *t*-tests (with Bonferroni correction) demonstrated a significant downregulation of sIPSC frequency for the AM630 ± PF3845 condition from control (0%; *p* < 0.001). For sIPSC amplitude (% of control), a three-way ANOVA demonstrated no significant effects and/or interactions (Figure 3E).

To assess mIPSCs, TTX was added to the bath to eliminate large-amplitude, action potential-dependent IPSCs. A three-way mixed ANOVA on mIPSC frequency (% of control) demonstrated similar effects as demonstrated on sIPSC frequency (% of control). A significant effect was noted for drug, *F*(1, 26) = 59.78, *p* < 0.001, with AM630 ± PF3845 treatment (*M* = −35.60, *SEM* = 3.82; *n* = 30/10) significantly downregulating sIPSC frequency (% of control) compared to AM630 condition (*M* = −9.81, *SEM* = 3.38; *n* = 30/10; *p* < 0.001; Figure 3F). No other effects and/or interactions were significant. Further, one-sample *t*-tests (with Bonferroni correction) indicated a significant downregulation of mIPSC frequency for AM630 and AM630 ± PF3845 treatments from control (0%; *p* = 0.028 and *p* < 0.001, respectively). For mIPSC amplitude (% of control), a three-way mixed ANOVA demonstrated no significant effects and/or interactions (Figure 3F). Overall, these data indicate that the CB_2_R antagonist AM630 was not able to block the downregulating effects of PF3845 on GABAergic neurotransmission and therefore PF3845′s inhibitory effects appear not to be regulated via CB_2_R-mediated mechanisms but involve CB_1_Rs.

### 3.4. Effects of PF3845 on Inhibitory GABAergic Neurotransmission Involve the Presence of Extracellular and Intracellular Calcium

**Figure 4:**
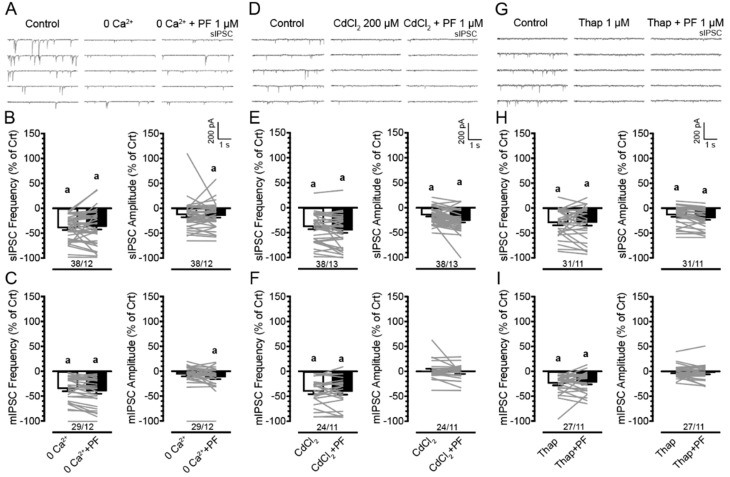
Effects of PF3845 on the frequency and amplitude of IPSCs (% of control) in mPFC neurons are blocked in the absence of extracellular and intracellular calcium. No significant effects were noted for sex and genotype on any sIPSC measure and are therefore collapsed across. (**A**) Representative traces show sIPSCs before and after 0 Ca^2+^ ± PF3845 (1 µM) bath application. (**B**) Removing extracellular calcium from the aCSF in the presence or absence of PF3845 (1 µM) significantly downregulated sIPSC frequency or amplitude (% of control; except for sIPSC amplitude at 0 Ca^2+^ condition) compared to control condition (before removal of 0 Ca^2+^, 0%). Importantly, the 0 Ca^2+^ ± PF3845 condition was not significantly different from 0 Ca^2+^, indicating PF3845 had no significant effect on sIPSC frequency or amplitude in the absence of extracellular calcium. (**C**) Similarly, mIPSC frequency or amplitude (% of control) was significantly downregulated by 0 Ca^2+^ ± PF3845 (except for mIPSC amplitude at 0 Ca^2+^) with PF3845 showing no further downregulation in the absence of extracellular calcium compared to the 0 Ca^2+^ condition. (**D**) Representative traces show sIPSCs before and after CdCl_2_ (200 µM) ± PF3845 (1 µM) bath application. (**E**) Application of CdCl_2_ significantly downregulated sIPSC frequency and amplitude (% of control) in the presence or absence of PF3845 compared to control condition (before CdCl_2_ treatment, 0%). No differences were noted between CdCl_2_ and CdCl_2_ ± PF3845 treatment conditions. (**F**) For mIPSCs, application of CdCl_2_ significantly downregulated mIPSC frequency (% of control) from control condition (0%), with CdCl_2_ ± PF3845 showing no further downregulation compared to the CdCl_2_ condition. No significant differences were noted for mIPSC amplitude (% of control). (**G**) Representative traces show sIPSCs before and after thapsigargin (1 µM) ± PF3845 (1 µM) bath application. (**H**) Application of thapsigargin significantly downregulated sIPSC frequency and amplitude (% of control) in the presence or absence of PF3845 compared to control condition (before thapsigargin treatment, 0%). No differences were noted between thapsigargin and thapsigargin + PF3845 treatment conditions, indicating that depletion of intracellular calcium stores blocked PF3845′s downregulating effects on sIPSC frequency and amplitude (% of control). (**I**) Similarly, mIPSC frequency was significantly downregulated by thapsigargin, with PF3845 showing no further downregulation compared to thapsigargin alone. No significant differences were noted for mIPSC amplitude (% of control). Data are percent of control data (mean ± SEM) collapsed across sex and genotype. Statistical significance was assessed by ANOVA followed by Bonferroni’s post hoc test and revealed no significant effects. One-sample *t*-tests with Bonferroni correction assessed significant changes from control condition (0%), ^a^ *p* < 0.05 vs. control (before treatment, 0%). In all panels, sample size is indicated as (cells/mice); please see Supplemental Table S1 for specific information on sample size for sex and genotype. 0 Ca^2+^, zero extracellular calcium; PF, PF3845; Thap, thapsigargin.

To understand the mechanisms by which PF3845 1 μM decreased GABAergic synaptic neurotransmission assessed by IPSCs, we examined the involvement of extracellular calcium be removing calcium from the bath solution (0 Ca^2+^, *n* = 6–14 neurons, 2–4 mice per group and sex; Figure 4A–C) and by adding a calcium channel blocker cadmium chloride to the bath solution calcium channels (CdCl_2_ 200 μM, *n* = 4–15 neurons, 2–4 mice per group and sex; Figure 4D–F). Involvement of intracellular calcium was assessed via thapsigargin (1 μM) application in the presence and absence of PF3845 (1 μM; Figure 4G–I). Three-way mixed ANOVAs were conducted on IPSC (% of control) with drug (2 levels: 0 Ca^2+^ or CdCl_2_ or thapsigargin, 0 Ca^2+^ + PF3845 or CdCl_2_ + PF3845 or thapsigargin + PF3845) as a within-subject factor and sex and genotype as between-subject factors (see Supplemental Result and Figures S3 and S4 for analysis and presentation of raw data). As no significant effects and/or interactions were noted for sex and/or genotype on any of the IPSC measures, data are presented combined for sex and genotype (Figure 4). Three-way mixed ANOVAs on sIPSC (frequency and amplitude; % of control) and mIPSC (frequency and amplitude; % of control) demonstrated no significant effects and/or interactions for any of the treatment conditions, indicating that PF3845′s ability to downregulate GABA release was abolished when external calcium was removed from the aCSF, or voltage-gated calcium channels were blocked with CdCl_2_. Similarly, depleting intracellular calcium stores via thapsigargin blocked the downregulating PF3845 effects on GABA release. Further, one-sample *t*-tests (with Bonferroni correction) indicated a significant downregulation of IPSC frequency and partly of IPSC amplitude from control (0%) for most of the treatment conditions (see Figure 4 for *M*, *SEM*, *n*), indicating the involvement of extracellular and intracellular calcium in GABAergic synaptic activity. Thus, the significant downregulation of action potential-dependent and action potential-independent GABA release (sIPSCs and mIPSCs) by PF3845 as well as GABAergic synaptic activity itself depend on extracellular and intracellular calcium.

### 3.5. Tat Transgenic Mice Display No Changes in CB_1_R, FAAH, and MAGL Protein Expression

**Figure 5:**
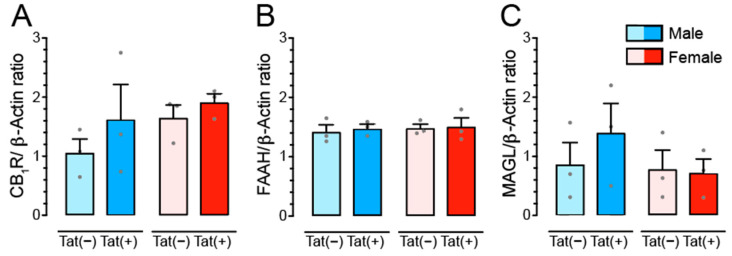
CB_1_R, FAAH, and MAGL protein expression levels are not altered by Tat and sex. Western blot analyses of protein expression levels for CB_1_R (**A**), FAAH (**B**), and MAGL (**C**) were assessed in the PFC of Tat transgenic mice. No significant effects were noted for any of the three measures. Data are eCB/β-Actin ratio data (mean ± SEM) separated by sex and genotype. Statistical significance was assessed by ANOVA. Sample size: *n* = 3 mice per group and sex.

To explore the impact of Tat induction on the endocannabinoid (eCB) system western blot analyses were conducted in the PFC of Tat transgenic mice (*n* = 3 mice per group and sex) to quantify cannabinoid type 1 receptor (CB_1_R) protein expression, protein expression of the AEA degrading enzyme FAAH, and protein expression of the 2-AG degrading enzyme MAGL. Two-way ANOVAs with sex and genotype as between subject-factors indicated no significant differences for any of the three measures (Figure 5).

### 3.6. Tat Transgenic Mice Display Altered Levels in AEA and Related Non-eCB Lipids in the PFC in a Sex and/or Tat-Dependent Manner

**Figure 6:**
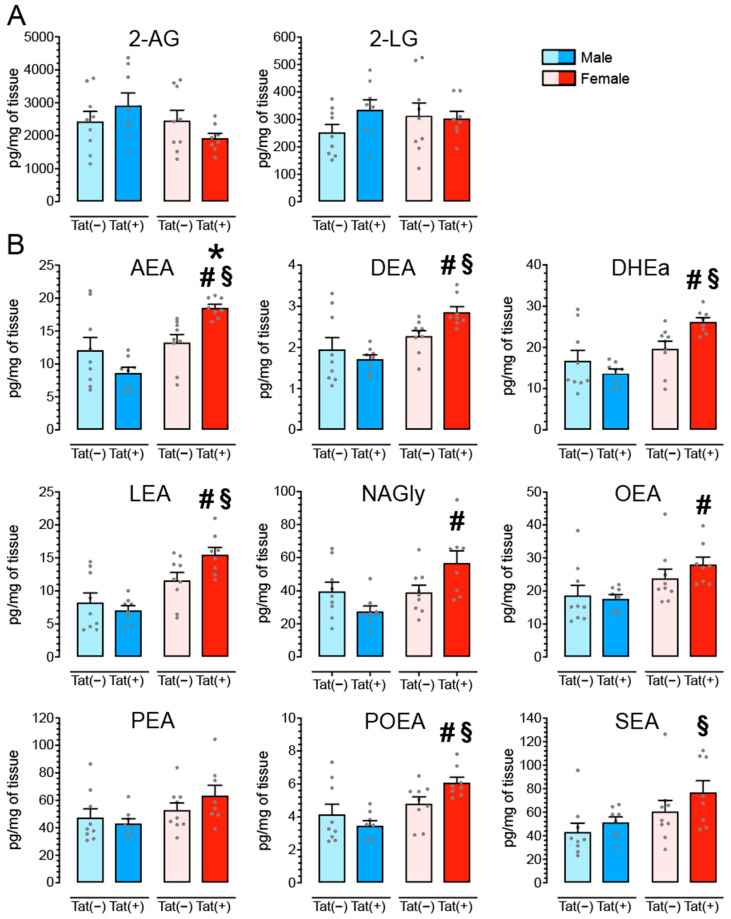
AEA and seven related non-eCB lipids are significantly upregulated in the PFC of female Tat(+) mice. Concentrations of AEA, 2-AG and other lipid molecules were assessed in the PFC of Tat transgenic mice using LC/MS/MS. Lipid concentrations were normalized to pg/mg of tissue. (**A**) No significant effects were noted for 2-AG or its related non-eCB lipid 2-LG. (**B**) For AEA and eight related non-eCB lipids, a significant sex and/or sex x genotype interaction was noted (see Table 1 for ANOVA results) with female Tat(+) mice demonstrating significant increased lipid concentrations in the PFC compared to one or more of the other three groups, except for PEA. Data of (non-)eCB concentration are expressed in pg/mg (mean ± SEM) separated by sex and genotype. Statistical significance was assessed by ANOVA followed by Bonferroni post hoc tests; * *p* < 0.05 vs. Tat(−) female, ^#^ *p* < 0.05 vs. Tat(+) male, ^§^ *p* < 0.05 vs. Tat(−) male. Sample size: *n* = 9, Tat(−) mice per sex; *n* = 8, Tat(+) mice per sex. Please see Supplemental Table S2 for the actual concentration values (mean ± SEM).

To determine the effects of Tat on eCB lipids we quantified levels of AEA, 2-AG, and nine related non-eCB lipids, in the PFC of Tat transgenic mice (*n* = 8–9 mice per group and sex; Table 1 and Figure 6). A one-way repeated ANOVA indicated significant differences between lipid concentrations (pg/mg), *F*(10, 330) = 241.1, *p* < 0.001, in the following order starting with the lowest: Docosatetraenyl ethanolamide (DEA; *M* = 2.20, *SEM* = 0.11; *n* = 34), *N*-palmitoleoyl ethanolamide (POEA; *M* = 4.62, *SEM* = 0.27; *n* = 34), Linoleoyl ethanolamide (LEA; *M* = 10.58, *SEM* = 0.79; *n* = 34), AEA (*M* = 13.10, *SEM* = 0.86; *n* = 34), Docosahexaenoyl ethanolamide (DHEa; *M* = 19.04, *SEM* = 1.16; *n* = 34), OEA (*M* = 22.02, *SEM* = 1.38; *n* = 34), NAGly (*M* = 40.79, *SEM* = 3.07; *n* = 34), PEA (*M* = 51.73, *SEM* = 3.04; *n* = 34), SEA (*M* = 57.68, *SEM* = 4.44; *n* = 34), 2-LG (*M* = 300.13, *SEM* = 17.76; *n* = 34), and 2-AG (*M* = 2438.81, *SEM* = 153.81; *n* = 34). All conditions, except for DHEa vs. OEA, NAGly vs. PEA, NAGly vs. SEA, PEA vs. SEA, significantly differ from each other (Bonferroni post hoc test’s, *p*’s < 0.001).

Two-way ANOVAs with sex and genotype as between subject-factors indicated a significant main effect of sex for AEA, DEA, DHEa, LEA, NAGly, OEA, PEA, POEA, and SEA as well as a significant sex x genotype interaction for AEA, DEA, DHEa, LEA, NAGly, and POEA (see ANOVA results in Table 1). No significant effects were noted for 2-AG or 2-LG. To explore the sex x genotype interactions further, Bonferroni post hoc tests demonstrated significant upregulation of AEA and seven non-eCB lipid concentrations for female Tat(+) mice compared to one or more of the other groups (see Figure 6; actual values for each lipid molecule are presented in Supplemental Table S2).

## 4. Discussion

The present study investigated the role of the eCB system in the HIV Tat transgenic mouse model on inhibitory GABAergic neurotransmission and how HIV Tat alters the eCB system, including cannabinoid receptor and enzyme protein expression as well as levels of AEA and 2-AG and related non-eCB lipids. Here we report significant alterations on GABAergic activity upon Tat exposure based on sex, with a suppression of inhibitory postsynaptic current (IPSCs) frequency and amplitude in Tat(+) male mPFC slices whereas Tat(+) females demonstrated an upregulation of IPSCs frequency. Interestingly, the effectiveness of PF3845 to suppress GABAergic activity in Tat transgenic mice was not altered by Tat induction or sex and involved CB_1_R related mechanisms that depended on extracellular and intracellular calcium. Additionally, our data indicated sex-dependent changes in levels of endogenous eCB and non-eCB lipids, in the PFC of Tat transgenic mice, for AEA, DEA, DHEa, LEA, NAGly, OEA, PEA, POEA, and SEA but not 2-AG or 2-LG, with sex effects being significantly altered by Tat induction for AEA, DEA, DHEa, LEA, NAGly, and POEA. No sex or Tat effects were noted for receptor or enzyme protein expression in the PFC of Tat transgenic mice. Overall, Tat alterations on inhibitory activity in the PFC depended on sex potentially due to sex-dependent changes of eCB and non-eCB lipid levels in the PFC upon chronic Tat induction; on the other hand, PF3845 suppression of inhibitory GABA activity was not altered by sex and/or Tat exposure and involved CB_1_R related mechanisms that depended on extracellular and intracellular calcium.

### 4.1. Inhibitory Neurotransmission Is Sex-Dependently Affected by Tat Exposure

Disturbance of neurotransmitter systems and circuits plays an important role in neuroHIV [84,85,86,87,88,89,90,91] and growing evidence indicates that inhibitory GABA neurotransmission is altered in the CNS of PWH [47,49]. A downregulation of the pre- and post-synaptic inhibitory GABA system was reported selectively in the frontal neocortex of PWH with neurocognitive impairments [47] and similar effects have been demonstrated in various mouse models of neuroHIV [31,40,43,45], even though an upregulation of GABA activity has also been reported depending on the brain region and synaptic marker involved [28,44]. The present study supports the downregulation of inhibitory synaptic activity in Tat(+) male mPFC slices including action potential-dependent and -independent release of GABA (sIPSCs and mIPSCs, respectively). As the frequency and amplitude of mIPSCs was reduced in male Tat(+) mice, decrease in GABAergic release could be due to pre- and post-synaptic mechanisms, i.e., the decrease in mIPSC frequency might be related to the inhibition of the vesicle release machinery in the presynaptic axon terminal (presynaptic inhibition) and a decrease of postsynaptic receptors could mimic the reduction in mIPSC amplitude (postsynaptic inhibition).

In contrast, a significant upregulation of inhibitory GABAergic neurotransmission was noted for female PFC Tat(+) slices, specifically for IPSC frequency but not amplitude, thus involving presynaptic but not postsynaptic inhibitory output. Existing data suggest that sex can significantly contribute to differences in synaptic neurotransmission which might play a role in sex-specific vulnerabilities to disease conditions, including schizophrenia, Alzheimer’s disease, and depression [92,93,94]. A recent study on schizophrenia reported a similar pattern as demonstrated in our current study, with males displaying reduced expression of GABAergic genes in the anterior cingulate cortex (ACC) of the medial PFC, and females demonstrating an overall increase of GABA gene expression [94]. Compensatory upstream mechanisms have been reported in schizophrenia that normalize GABA concentrations from an initial downregulation to an increase in synaptic activity of GABA [95]. This is interesting as the increased inhibitory synaptic activity in female mice could be attributed to a compensatory effect, since increases in excitatory synaptic activity via the glutamate system have been frequently reported in PWH and neuroHIV models [32,96,97]. An additional argument for a potential compensatory response of increased GABAergic neurotransmission in the PFC of female Tat(+) mice is the selected upregulation of AEA and related non-eCB lipids found in the PFC of Tat(+) females in the current study (Figure 6, discussed further below).

### 4.2. PF3845 Decreases GABAergic Neurotransmission Independent of Sex and Genotype via CB_1_R-Related Mechanisms

It is well known that cannabinoids function as retrograde messengers that activate presynaptic CB_1_Rs and reduce inhibitory GABA release via inhibition of calcium channels [98,99]. Nevertheless, effects of AEA and FAAH enzyme inhibition on GABAergic synaptic activity are variable depending on the brain region involved [45,100]. Our present study supports a downregulation of GABAergic synaptic activity in the PFC by the FAAH enzyme inhibitor PF3845 via CB_1_R-related mechanisms that is dependent on extracellular and intracellular calcium. Whereas CB_1_R antagonist SR141716A prevented the PF3845-induced decrease in GABA release, AM630, a CB_2_R antagonist, failed to block the PF3845-induced effects on GABA neurotransmission. Similarly, removing extracellular calcium prevented PF3845-induced decrease in GABA release and was also blocked by using a voltage-gated calcium channel subtype. It has been shown previously that the sensitivity of GABA release depends on extracellular calcium with the involvement of different voltage-gated calcium channel subtypes [101]. Further, it should be noted that removal of intracellular calcium also blocked the PF3845-induced effects on GABA neurotransmission, suggesting that intracellular calcium stores play a role in PF3845-mediated inhibition of GABA release. Studies have demonstrated that cannabinoids produce neuroprotection by reducing intracellular calcium release from ryanodine-sensitive stores [102]. Overall, it is suggested that PF3845 activates CB_1_Rs presynaptically and downregulates GABA release via the well-known CB_1_R-mediated retrograde synaptic signaling mechanism by inhibiting Ca^2+^ channels and intracellular calcium stores [100,102,103,104,105,106].

An unexpected finding was that PF3845′s inhibitory effects on GABAergic synaptic activity was not altered by sex and Tat exposure. Sex- and disease-dependent effects have been reported for FAAH enzyme inhibitors (e.g., PF3845, URB597) in vivo, including producing anti-anxiety effects under stressful conditions without affecting control animals [107], and improving cognitive function in male but not female rats [108]. Further, recent studies demonstrated Tat-induced impairment or enhancement of cannabinoid-mediated inhibition of transmitter release, depending on the brain region involved [32,48]. Nevertheless, previous reported effects of Tat on the cannabinoid system were specific to glutamatergic neurotransmission and no effects were noted for the inhibitory GABAergic system [48]. Thus, whether the induced differential response by PF3845 in the presence of Tat is specific to excitatory and not inhibitory neurotransmission and how PF3845 would affect synaptic activity in the context of neuroHIV in vivo, especially when given chronically, remains uncertain but is a compelling area for further investigation.

### 4.3. Tat Exposure Significantly Alters AEA and Related Non-eCB Lipids in the PFC of Female Mice but Not Male Mice

Potential effects of neuroHIV on the eCB system have been reviewed recently [53,55,56]. The present study found no differences for CB_1_R, FAAH, and MAGL protein expression levels in the PFC of Tat transgenic mice, potentially due to the low sample size that increased the likelihood of type II error. Variable effects on CB_1_R protein expression levels have been reported in the context of neuroHIV [20,32,71,72,73,109,110]. A previous study from our laboratory reported neuronal CB_1_R upregulation in the infralimbic region of stressed female Tat(+) mice compared to their control Tat(−) females via immunohistochemistry, which was associated with behavioral deficits in an inhibitory control task [32]. The association that CB_1_R expression upregulates with increased cognitive impairments has been supported in a recent immunohistochemistry study in PWH, where CB_1_R levels in neurons in the frontal lobe significantly increased with increasing degree of cognitive impairment in PWH [73]. In comparison to cannabinoid receptor levels, much less is known about changes of expression levels of eCB degrading enzymes in the context of neuroHIV, including FAAH and MAGL enzymes that degrade AEA and 2-AG, respectively [58,59,60]. In recent studies, FAAH was found to be overexpressed in perivascular astrocytes of cortical SIV tissue samples [73] and neuritic plaque-associated astrocytes in Alzheimer’s disease brains [111]. Noteworthy, even though the present study found no differences in FAAH enzyme expression, female Tat(+) mice demonstrated upregulated levels of AEA and related non-eCB lipids in the PFC (Figure 6). As FAAH is a degrative enzyme for AEA, it would be beneficial to assess cell-specificity of FAAH expression in a follow up study as it seems to be specifically localized in astrocytes and downregulation of cell-specific FAAH expression could potentially contribute to the upregulated levels of AEA in the PFC of female Tat(+) mice. Thus, immunohistochemistry and other molecular tests, such as assessing mRNA expression via polymerase chain reaction, would be important methods to complement Western blot data and should be included in future studies.

The selected upregulation of AEA and related non-eCB lipids specifically in the PFC of Tat(+) females but not male mice is an interesting finding and parallels a previous study that demonstrated upregulated CB_1_R expression levels in female Tat(+) mice which also showed inhibitory control deficits [32]. In general, HIV literature finds a higher vulnerability of HAND in women [112,113,114,115,116] even though it appears that HIV+ men and women may have different cognitive profiles that are domain specific [117,118,119]. Additional studies are needed to clarify the difference in HAND pathology between sexes and the differential role that sex plays on the eCB system in HAND. Further, the finding of upregulated eCB levels in Tat(+) females needs to be investigated more in detail, specifically in the light of female Tat(+) mPFC slices demonstrating increased GABAergic synaptic activity. Not a lot of work has looked at changes in eCB lipids in the CNS of PWH and it is unknown if eCB ligands, including AEA or 2-AG, are increased in the PWH brain. Recent in vitro studies from our laboratory found no significant Tat-induced changes in eCB levels but AEA and related non-eCB lipids (PEA and/or OEA) were trending towards an upregulation upon Tat exposure [19,70]. It is well known that AEA is synthesized and released on demand to repair and provide homeostasis; therefore, its endogenous tone in the brain can change depending on the insult that happens. Multiple studies have demonstrated that Tat induces neuroinflammation, neuronal injury, and excitotoxicity in various brain regions, including the PFC [19,32,43,45,70,97]; thus, upregulation of AEA in the PFC of Tat(+) females could be a mechanism to counteract Tat-induced neurodegenerative changes. However, it is known that AEA is a partial agonist for CB_1_R [120] and it has been widely demonstrated that activation of CB_1_R reduces rather than facilitates GABAergic synaptic activity [98,99]. Similarly, AEA can activate transient receptor potential vanilloid type 1 (TRPV1) receptors [121,122,123], that also have been shown to decrease GABAergic neurotransmission in the mPFC of mice [124]. Noteworthy, as demonstrated in the present study, structurally related lipids to AEA, including PEA, OEA, and NAGly, which do not activate cannabinoid receptors, are often altered in parallel with changes in AEA [125,126,127,128]. PEA and OEA activate other G-protein-coupled receptors, including GPR55 and GPR119 [129,130], and other receptor systems including peroxisome proliferator-activated alpha receptors PPAR-α [131,132] and TRPV1 [133,134,135]. The effects of PEA on GABA activity have been demonstrated previously, in which PEA enhanced GABA transmission in the striatum by acting on GPR55 [136]. Further, NAGly, which is an endogenous lipid derived from AEA but lacking affinity for the CB_1_R, has been demonstrated to activate GPR18 [137,138,139]. GPR18s are specifically found on microglia and NAGly-GPR18 signaling has been shown to exert protective effects against excitotoxic neuronal damage [140]. We speculate that the Tat-induced changes for AEA and related eCB lipids, including DEA and NAGly, in the female PFC, as well as the upsurges in GABAergic neurotransmission, represent a homeostatic response to the insult caused by Tat induction. The activation of selective PPARs, GPR55, and/or GPR18 by these endogenous ligands may play a role in upregulating GABAergic synaptic activity. In preclinical disease models it has been demonstrated that the dysregulation of the eCB system contributes to changes in inhibitory synaptic activity in various brain regions [136,141,142]. However, the causal relationship between the eCB system and GABA activity is not clear. A recent study demonstrated that long-term increases of GABA release were responsible for driving changes in eCB lipid levels [143]. The exact mechanisms underlying the findings in the present study are not investigated here and require further investigation specifically the role that sex plays in this context.

## 5. Conclusions

In the present study, we evaluated the role of the eCB system in a neuroHIV mouse model (i.e., female and male HIV-1 Tat transgenic mice) by first assessing potential protective effects elicited by a FAAH enzyme inhibitor PF3845 on inhibitory synaptic activity in mPFC brain slices (neurotransmission; Figure 2), and second by examining changes in receptor, enzyme, and lipid expression levels of the eCB system in PFC tissue (Figure 5 and Figure 6). Although PF3845 elicited its effects independent of sex and Tat with inhibiting GABA neurotransmission for all groups in a similar manner via CB_1_R-related mechanisms, significant sex differences were noted for the protein and lipid signature of the eCB system as well as the inhibitory synaptic activity depending on Tat exposure. Specifically, female Tat(+) animals demonstrated upregulated AEA and non-eCB lipid levels, which potentially contribute to a compensatory response in the GABAergic system. Overall, our results suggest that the eCB system is significantly altered under conditions of HAND in a sex-dependent manner which should be taken into account when considering eCB treatments as potential therapeutic options against neuroHIV pathogenesis.

## Figures and Tables

**Table 1:** ANOVA results for sex and genotype on eCB and other lipid signaling molecule levels (pg/mg) in the prefrontal cortex of HIV Tat transgenic mice ^a^.

Lipids	Sex Effect		Genotype Effect		Sex x Genotype	
pg/mg	*F*_1, 30_	*p*	*F*_1, 30_	*p*	*F*_1, 30_	*p*
2-AG	2.7	0.114	<1.0	0.939	3.0	0.096
2-LG	<1.0	0.697	1.0	0.318	1.6	0.211
AEA	**18.3**	**<0.001**	<1.0	0.493	**11.5**	**0.002**
DEA	**15.6**	**<0.001**	<1.0	0.360	**4.9**	**0.035**
DHEa	**18.4**	**<0.001**	1.0	0.325	**7.0**	**0.013**
LEA	**25.6**	**<0.001**	1.3	0.261	**4.7**	**0.038**
NAGly	**7.5**	**0.010**	<1.0	0.605	**8.0**	**0.008**
OEA	**9.7**	**0.004**	<1.0	0.537	1.1	0.307
PEA	**5.0**	**0.033**	<1.0	0.592	1.7	0.208
POEA	**13.6**	**<0.001**	<1.0	0.509	**5.0**	**0.033**
SEA	**6.8**	**0.014**	2.2	0.149	<1.0	0.630

^a^ Levels of eCB and non-eCB lipids (*n* = 8–9 mice per group) in the prefrontal cortex of Tat transgenic mice. Two-way ANOVAs were conducted with sex and genotype as between-subject factors. *F*-values and *p*-values are presented from ANOVA results. Bold values indicate significance. Data (expressed as mean ± SEM in pg/mg) with Bonferroni post hoc correction for multiple comparisons as necessary are represented in Figure 6.

## Data Availability

Data supporting reported results will be shared with other investigators upon request.

## Supplementary Materials

The following supporting information can be downloaded at: https://www.mdpi.com/article/10.3390/cells11050857/s1, Table S1: Sample size (cell/mice) information based on sex and genotype for all treatment conditions, Table S2: Levels of eCB and non-eCB lipids in the prefrontal cortex of HIV Tat transgenic mice expressed in pg/mg as mean and SEM, Figure S1: PF3845 significantly induces a decrease in the IPSC frequency in mPFC neurons independent of Tat induction and sex, Figure S2: PF3845 effects on IPSCs in mPFC neurons are blocked by CB_1_R antagonist SR141716A but not CB_2_R antagonist AM630, Figure S3: Effects of PF3845 on IPSCs in mPFC neurons are blocked in the absence of extracellular calcium, Figure S4: Depletion of intracellular calcium stores via thapsigargin affects IPSCs in mPFC neurons depending on sex and Tat induction and blocks PF3845′s downregulating effects similarly across groups.
