# Supplementary material for: Inhibitory Neurotransmission Is Sex-Dependently Affected by Tat Expression in Transgenic Mice and Suppressed by the Fatty Acid Amide Hydrolase Enzyme Inhibitor PF3845 via Cannabinoid Type-1 Receptor Mechanisms
Source: Cells. 2022 Mar 2;11(5):857. doi: 10.3390/cells11050857 (PMC8909692; doi:10.3390/cells11050857)
Supplement: Supplementary file 1 [file cells-11-00857-s001.zip › cells-1517213-supplementary.pdf]

Supplemental Material

Supplemental Results

PF3845-induced downregulation (PF3845 Δ change in %) of inhibitory GABAergic neurotransmission occurs independent of Tat induction and sex. To explore the effects of cannabinoids in the presence of Tat on sIPSCs and mIPSCs, we performed patch-clamp recordings on mPFC pyramidal neurons in Tat transgenic mice with bath application of PF3845 (1 μM, n = 8-18 neurons, 3-5 mice per group and sex). Data are presented as PF3845-induced change (Δ) from baseline (0%, **Supplemental Figure S1**). For sIPSC frequency, an overall one-sample t-test demonstrated a significant inhibitory effect of PF3845, $t(59) = -9.86$, $p < 0.001$, with separate one-sample t-tests (with using Bonferroni correction) demonstrating PF3845-induced percent inhibition for all groups [male Tat(-): $M = -42.54$, $SEM = 6.70$, n = 18/5, $p = 0.004$; male Tat(+):$M = -33.16$, $SEM = 8.18$, n = 17/5, $p < 0.001$; female Tat(-): $M = -43.49$, $SEM = 11.25$, n = 10/3, $p = 0.016$; female Tat(+): $M = -38.64$, $SEM = 7.31$, n = 15/5, $p < 0.001$; **Figure S1A**]. A two-way ANOVA revealed no significant Sex and/or Genotype effects. For percent sIPSC amplitude an overall one-sample t-test demonstrated a significant inhibitory effect of PF3845, $t(59) = -2.54$, $p = 0.014$, with separate one-sample t-tests demonstrating no significant PF3845-induced percent inhibition for any group (**Figure S1B**). A two-way ANOVA revealed no significant Sex and/or Genotype effects.

To assess mIPSCs, TTX was added to the bath to eliminate large-amplitude, action potential-dependent IPSCs. An overall one-sample t-test on mIPSC frequency demonstrated a significant inhibitory effect of PF3845, $t(54) = -9.07$, $p < 0.001$, with separate one-sample t-tests (with using Bonferroni correction) demonstrating PF3845-induced percent inhibition for all groups [male Tat(-): $M = -34.48$, $SEM = 8.61$, n = 16/5, $p = 0.032$; male Tat(+): $M = -37.97$, $SEM = 6.98$, n = 15/5, $p < 0.001$; female Tat(-): $M = -40.05$, $SEM = 11.03$, n = 8/3, $p = 0.032$; female Tat(+): $M = -32.50$, $SEM = 6.66$, n = 16/5, $p < 0.001$; **Figure S1C**]. A two-way ANOVA revealed no significant Sex and/or Genotype effects. For mIPSC amplitude an overall one-sample t-test demonstrated a significant inhibitory effect of PF3845, $t(54) = -2.99$, $p = 0.004$, with separate one-sample t-tests (with using Bonferroni correction) demonstrating no significant PF3845-induced percent inhibition for any group (**Figure S1D**). A two-way ANOVA revealed no significant Sex and/or Genotype effects. Thus, PF3845 inhibits GABAergic neurotransmission overall presynaptically independent of Tat induction or sex.

PF3845's inhibitory effects on GABAergic neurotransmission are mediated by CB_1Rs but not CB_2Rs. We have shown that PF3845 bath application decreases GABAergic neurotransmission independent of Tat induction and sex but the underlying CBR-related mechanisms are not clear. Thus, we examined the FAAH enzyme inhibitor PF3845 on IPSCs on mPFC neurons in Tat transgenic mPFC brain slices with bath application of the CB_1R antagonist SR141716A (1 μM; n = 6-14 neurons, 2-3 mice per group and sex; **Supplemental Figure S2A-B**) and the CB_2R antagonist AM630 (1 μM; n = 7-8 neurons, 2-3 mice per group and sex; **Supplemental Figure S2C-D**). Three-way mixed ANOVAs were conducted with Drug (3 levels: Control, CBR antagonist, CBR antagonist + PF3845) as a within-subjects factor and Sex and Genotype as between-subjects factors. As no significant effects and/or interactions were noted for Sex and/or Genotype on any of the IPSC measures, data are presented combined for Sex and Genotype (**Figure S2,** see **Supplemental Table S1** for specific information on sample size for group and sex).

Blocking CB_1Rs with SR141716A (1 μM) bath application. Three-way mixed ANOVAs on sIPSC (frequency and amplitude) and mIPSC (frequency and amplitude) demonstrated no significant effects and/or interactions (**Figure S2A, S2B**). These data indicate that the CB_1R antagonist SR141716A (1 μM) by itself had no significant effect on action potential-dependent and action potential-independent GABA release, and further that SR141716A was able to block the downregulating effects of PF3845 on GABAergic neurotransmission, suggesting PF3845's inhibitory effects involve CB_1R-mediated mechanisms.

Blocking CB_2Rs with AM630 (1 μM) bath application. A three-way mixed ANOVA on sIPSC frequency demonstrated a significant effect for Drug, $F(2, 54) = 10.88$, $p < 0.001$, with AM630 ± PF3845 treatment (M = 1.81, SEM = 0.22; n = 31/10) significantly downregulating sIPSC frequency compared to control condition before treatment exposure (M = 2.49, SEM = 0.27; n = 31/10; p = 0.004) and compared to AM630 condition (M = 2.44, SEM = 0.30; n = 31/10; p = 0.002; **Figure S2C**), indicating AM630 pretreatment was not able to block the downregulating effect of PF3845 on sIPSC frequency. No other effects and/or interactions were significant. For sIPSC amplitude, a three-way ANOVA demonstrated no significant effects and/or interactions (**Figure S2C**).

To assess mIPSCs, TTX was added to the bath to eliminate large-amplitude, action potential-dependent IPSCs. A three-way mixed ANOVA on mIPSC frequency demonstrated similar effects as demonstrated on sIPSC frequency. A significant effect was noted for Drug, $F(2, 52) = 25.05$, $p < 0.001$, with AM630 ± PF3845 treatment (M = 1.23, SEM = 0.16; n = 30/10) significantly downregulating sIPSC frequency compared to control condition (M = 1.89, SEM = 0.23; n = 30/10; $p < 0.001$) and AM630 condition (M = 1.76, SEM = 0.23; n = 30/10; $p < 0.001$; **Figure S2D**). No other effects and/or interactions were significant. For mIPSC amplitude, a three-way mixed ANOVA demonstrated a significant effect for Drug, $F(2, 52) = 5.64$, $p = 0.006$, with AM630 ± PF3845 treatment (M = 21.27, SEM = 1.26; n = 30/10) significantly downregulating mIPSC amplitude compared to control condition before any treatment (M = 23.46, SEM = 1.51; n = 30/10; p = 0.043; **Figure S2D**). These data indicate that the CB_2R antagonist AM630 was not able to block the downregulating effects of PF3845 on GABAergic neurotransmission and therefore PF3845's inhibitory effects appear not to be regulated via CB_2R-mediated mechanisms but involve CB_1Rs.

Effects of PF3845 on inhibitory GABAergic neurotransmission involve the presence of extracellular calcium. To understand the mechanisms by which PF3845 1 μM decreased GABAergic synaptic neurotransmission assessed by IPSCs, we examined the involvement of extracellular calcium be removing calcium from the bath solution (0 Ca^{2+}, n = 6-14 neurons, 2-4 mice per group and sex**, Supplemental Figure S3A-B**) and by adding a calcium channel blocker cadmium chloride to the bath solution calcium channels ($CdCl_2$ 200 μM, n = 4-15 neurons, 2-4 mice per group and sex**, Supplemental Figure S3C-D**). Three-way mixed ANOVAs were conducted with Drug (3 levels: Control, 0 Ca^{2+} or $CdCl_2$, 0 Ca^{2+} + PF3845 or $CdCl_2$ + PF3845) as a within-subjects factor and Sex and Genotype as between-subjects factors. As no significant effects and/or interactions were noted for Sex and/or Genotype on any of the IPSC measures, data are presented combined for Sex and Genotype (**Figure S3**).

Removing extracellular calcium from the bath solution (0 Ca^{2+} condition). A three-way mixed ANOVA on sIPSC frequency demonstrated a significant effect for Drug, $F(2, 68) = 31.70$, $p < 0.001$, with 0 Ca^{2+} significantly downregulating sIPSC frequency for the 0 Ca^{2+} condition (M = 1.99, SEM = 0.27; n = 38/12; $p < 0.001$) and 0 Ca^{2+} ± PF3845 (M = 2.16, SEM = 0.33; n = 38/12; $p < 0.001$) compared to control condition before treatment exposure (M = 3.29, SEM = 0.37; n = 38/12; **Figure S3A**), indicating the involvement of external calcium in GABAergic synaptic activity. More importantly, in the absence of external calcium, no significant PF3845 effect was noted on sIPSCs frequency compared to zero extracellular calcium alone. Similarly, a three-way mixed ANOVA on sIPSC amplitude demonstrated a significant effect for Drug, $F(2, 68) = 9.51$, $p < 0.001$, with 0 Ca^{2+} significantly downregulating sIPSC amplitude for the 0 Ca^{2+} condition (M = 23.22, SEM = 1.41; n = 38/12; p = 0.022) and 0 Ca^{2+} ± PF3845 (M = 22.64, SEM = 1.19; n = 38/12; p = 0.005) compared to control condition before treatment exposure (M = 28.47, SEM = 2.12; n = 38/12; **Figure S3A**). Additionally, in the absence of external calcium, no significant PF3845 effect was noted on sIPSCs amplitude compared to zero extracellular calcium alone.

For mIPSCs, similar effects were noted. A three-way mixed ANOVA on mIPSC frequency and amplitude demonstrated a significant effect for Drug, $F(2, 50) = 29.27$, $p < 0.001$, with zero external calcium significantly downregulating mIPSC frequency for the 0 Ca^{2+} condition (M = 1.62, SEM = 0.33; n = 29/12; $p < 0.001$) and 0 Ca^{2+} ± PF3845 (M = 1.51, SEM = 0.29; n = 29/12; $p < 0.001$) compared to control condition before treatment exposure (M = 2.27, SEM = 0.38; n = 29/12; **Figure S3B**). In the absence of external calcium, no

significant PF3845 effect was noted on mIPSCs frequency compared to zero extracellular calcium alone. Similarly, a three-way mixed ANOVA on mIPSC amplitude demonstrated a significant effect for Drug, $F(2, 50) = 6.24$, $p = 0.004$, with 0 Ca^{2+} significantly downregulating sIPSC amplitude for the 0 Ca^{2+} ± PF3845 treatment ($M = 21.20$, $SEM = 1.68$; $n = 29/12$; $p = 0.003$) compared to control condition before treatment exposure ($M = 23.91$, $SEM = 1.66$; $n = 29/12$), with 0 Ca^{2+} + PF3845 not significantly differing from the 0 Ca^{2+} condition (**Figure S3B**). Thus, the significant downregulation of action potential-dependent and action potential-independent GABA release (sIPSCs and mIPSCs) by PF3845 was abolished when no external calcium was present in the aCSF.

Blocking voltage-gated calcium channels with $CdCl_2$ (200 μM). A three-way mixed ANOVA on sIPSC frequency demonstrated a significant effect for Drug, $F(2, 68) = 51.95$, $p < 0.001$, with $CdCl_2$ significantly downregulating sIPSC frequency for the $CdCl_2$ condition ($M = 2.17$, $SEM = 0.30$; $n = 38/13$; $p < 0.001$) and $CdCl_2$ ± PF3845 ($M = 2.03$, $SEM = 0.32$; $n = 38/13$; $p < 0.001$) compared to control condition before treatment exposure ($M = 3.40$, $SEM = 0.37$; $n = 38/13$; **Figure S3C**). More importantly, when blocking calcium channels with $CdCl_2$, no significant PF3845 effect was noted on sIPSCs frequency compared to $CdCl_2$ treatment alone. For sIPSC amplitude, a three-way ANOVA demonstrated a significant effect for Drug, $F(2, 68) = 38.14$, $p < 0.001$, with $CdCl_2$ significantly downregulating sIPSC amplitude for the $CdCl_2$ condition ($M = 20.44$, $SEM = 0.92$; $n = 38/13$; $p < 0.001$) and $CdCl_2$ ± PF3845 ($M = 17.71$, $SEM = 0.98$; $n = 38/13$; $p < 0.001$) compared to control condition before treatment exposure ($M = 24.59$, $SEM = 1.11$; $n = 38/13$; **Figure S3C**). Interestingly, whereas no significant PF3845 effect on the frequency of sIPSCs was noted in the presence of $CdCl_2$, PF3845 induced a significant decrease in the amplitude of mIPSCs in the presence of $CdCl_2$ compared to the $CdCl_2$ condition, indicating other channels and receptors that are involved in Ca^{2+} influx contribute to PF3845-induced effects on action potential-dependent GABAergic neurotransmission postsynaptically.

For mIPSCs, a three-way mixed ANOVA demonstrated a significant effect for Drug, $F(2, 40) = 36.02$, $p < 0.001$, with $CdCl_2$ significantly downregulating mIPSC frequency for the $CdCl_2$ condition ($M = 1.05$, $SEM = 0.19$; $n = 24/11$; $p < 0.001$) and $CdCl_2$ ± PF3845 ($M = 1.05$, $SEM = 0.20$; $n = 24/11$; $p < 0.001$) compared to control condition before treatment exposure ($M = 1.65$, $SEM = 0.25$; $n = 24/11$; **Figure S3D**). When blocking calcium channels with $CdCl_2$, no significant PF3845 effect was noted on mIPSCs frequency compared to $CdCl_2$ treatment alone. For sIPSC amplitude, a three-way ANOVA revealed no significant effects. Thus, the significant downregulation of GABA release (sIPSCs and mIPSCs) by PF3845 was abolished when calcium channels were blocked with $CdCl_2$, except for action potential-dependent GABA release postsynaptically (sIPSC amplitude) in which case other channels and receptors that are involved in Ca^{2+} influx could potentially contribute to PF3845-induced effects.

Depleting intracellular calcium stores affects GABAergic neurotransmission depending on Tat induction and sex and blocks PF3845 effects similarly across groups. To explore the effects of intracellular calcium on GABA release in the context of Tat induction and FAAH enzyme inhibition, we examined the involvement of intracellular calcium via thapsigargin (1 μM) application in the presence and absence of PF3845 (1 μM, **Supplemental Figure S4**). Three-way mixed ANOVAs were conducted with Drug (3 levels: Control, Thapsigargin, Thapsigargin + PF3845) as a within-subjects factor and Sex and Genotype as between-subjects factors. For sIPSC frequency, a three-way mixed ANOVA demonstrated a significant effect for Drug, $F(2, 54) = 22.21$, $p < 0.001$, with thapsigargin significantly downregulating sIPSC frequency before PF3845 treatment ($M = 2.10$, $SEM = 0.33$; $n = 31/11$; $p = 0.002$) and after PF3845 application ($M = 2.20$, $SEM = 0.43$; $n = 31/11$; $p < 0.001$) compared to control condition before thapsigargin exposure ($M = 2.86$, $SEM = 0.43$; $n = 31/11$), however no significant PF3845 effect was noted on sIPSCs frequency in the presence of thapsigargin compared to the thapsigargin alone condition (**Figure S4B**). Importantly, the significant main effect of Drug was significantly altered by Sex (Drug x Sex interaction; $F(2, 54) = 4.86$, $p = 0.011$) and by Sex and Genotype (Drug x Sex x Genotype interaction; $F(2, 54) = 4.39$, $p = 0.017$; **Figure S4B**). Thus, separate one-way ANOVAs were conducted to explore the effects of Drug on sIPSC frequency in each group. Whereas, no significant Drug effect was demonstrated for Tat(−) males or Tat(+) females, a significant Drug

effect was noted for Tat(+) males, $F(2, 12) = 8.38$, $p = 0.005$, and Tat(−) females, $F(2, 10) = 17.78$, $p = 0.001$, with thapsigargin significantly downregulating sIPSC frequency for the thapsigargin condition [Tat(+) males: $M = 0.97$, $SEM = 0.31$; $n = 7/3$; $p = 0.053$; Tat(−) females: $M = 1.28$, $SEM = 0.42$; $n = 7/2$; $p = 0.038$] and thapsigargin ± PF3845 treatment [Tat(+) males: $M = 0.88$, $SEM = 0.20$; $n = 7/3$; $p = 0.051$; Tat(−) females: $M = 1.10$, $SEM = 0.47$; $n = 7/2$; $p = 0.013$] compared to control condition before treatment exposure [Tat(+) males: $M = 1.57$, $SEM = 0.38$; $n = 7/3$; Tat(−) females: $M = 2.58$, $SEM = 0.64$; $n = 7/2$; **Figure S4B**]. No significant PF3845 effects were noted on sIPSCs frequency in the presence of thapsigargin compared to the thapsigargin alone condition in any group, indicating the PF3845 effects were abolished in the presence of thapsigargin for all groups. Lastly, the three-way mixed ANOVA demonstrated a significant Sex x Genotype interaction on sIPSC frequency, $F(1, 27) = 7.59$, $p = 0.010$, with increased sIPSC frequency for Tat(+) females ($M = 4.35$, $SEM = 1.07$; $n = 8/3$; $p = 0.017$) compared to Tat(+) males ($M = 1.14$, $SEM = 0.29$; $n = 7/3$; **Figure S4B**). Thus, overall thapsigargin significantly downregulated action potential-dependent GABAergic neurotransmission (sIPSCs) dependening on sex and genotype and blocked the downregulating PF3845 effects on GABA release similar across groups.

For sIPSC amplitude, a three-way ANOVA on sIPSC amplitude revealed a significant effect for Drug, $F(2, 54) = 24.74$, $p < 0.001$, a significant Drug x Genotype interaction, $F(2, 54) = 7.78$, $p = 0.001$, and a significant Sex x Genotype interaction, $F(1, 27) = 9.72$, $p = 0.004$ (**Figure S4C**). Separate one-way ANOVAs demonstrated a significant Drug effect for Tat(−) females, $F(2, 12) = 10.86$, $p = 0.002$, and Tat(−) males, $F(2, 16) = 9.64$, $p = 0.002$, with thapsigargin significantly downregulating sIPSC amplitude for the thapsigargin ± PF3845 treatment [Tat(−) females: $M = 13.84$, $SEM = 0.85$; $n = 7/2$; $p = 0.031$; Tat(−) males: $M = 20.56$, $SEM = 2.69$; $n = 9/3$; $p = 0.036$] compared to control [Tat(−) females: $M = 25.13$, $SEM = 3.37$; $n = 7/2$; Tat(−) males: $M = 26.84$, $SEM = 3.09$; $n = 9/3$; **Figure S4C**]. No significant PF3845 effects were noted on sIPSCs amplitude in the presence of thapsigargin compared to the thapsigargin alone condition in any group, indicating the PF3845 effects were abolished in the presence of thapsigargin for all groups.

For mIPSCs, a three-way mixed ANOVA on mIPSC frequency demonstrated a significant effect for Drug, $F(2, 46) = 22.91$, $p < 0.001$, with thapsigargin downregulating mIPSC frequency for the thapsigargin condition ($M = 1.41$, $SEM = 0.29$; $n = 27/11$) and thapsigargin ± PF3845 treatment ($M = 1.40$, $SEM = 0.25$; $n = 27/11$) compared to control condition before treatment exposure ($M = 1.78$, $SEM = 0.32$; $n = 27/11$; **Figure S4D**). No significant PF3845 effects were noted on mIPSCs frequency in the presence of thapsigargin compared to the thapsigargin alone condition in any group. Further a significant Sex x Genotype interaction was noted on mIPSC frequency, $F(1, 23) = 10.89$, $p = 0.003$, with increased mIPSC frequency for Tat(+) females ($M = 2.85$, $SEM = 0.75$; $n = 7/3$) compared to Tat(−) females ($M = 0.60$, $SEM = 0.18$; $n = 5/2$, $p = 0.029$) and Tat(+) males ($M = 0.89$, $SEM = 0.13$; $n = 10/3$, $p = 0.023$; **Figure S4C**).

For mIPSC amplitude a three-way ANOVA on mIPSC amplitude revealed a significant effect for Genotype, $F(1, 23) = 8.46$, $p = 0.008$ [Tat(−): $M = 16.06$, $SEM = 1.59$; $n = 10/5$; Tat(+): $M = 20.37$, $SEM = 1.26$; $n = 17/6$], that was significantly altered by sex [Sex x Genotype interaction: $F(1, 23) = 14.67$, $p = 0.001$], with increased mIPSC amplitude for Tat(+) females ($M = 23.96$, $SEM = 1.23$; $n = 7/3$) compared to Tat(−) females ($M = 12.73$, $SEM = 0.16$; $n = 5/2$, $p = 0.001$) and Tat(+) males ($M = 17.85$, $SEM = 1.55$; $n = 10/3$, $p = 0.040$; **Figure S4D**). No other effects and/or interactions were significant. Overall thapsigargin significantly downregulated GABAergic neurotransmission (sIPSCs and mIPSCs) in male Tat(+) mice and female Tat(−) mice but not in the other two groups. Further, depleting intracellular calcium stores via thapsigargin blocked the downregulating PF3845 effects on GABA release similar across groups.

Supplemental Tables

Tables

Table S1. Sample size (cell/mice) information based on Sex and Genotype for all treatment conditions *.

Treatment	Genotype	sIPCSs			mIPCSs		
		**Male**	**Female**	**Total**	**Male**	**Female**	**Total**
Control[$]	Tat(−)	18/5	10/3	60/18	16/5	8/3	55/18
	Tat(+)	17/5	15/5		15/5	16/5	
SR141716A (1 µM)	Tat(−)	11/3	14/2	48/10	6/3	7/2	30/10
	Tat(+)	12/3	11/2		10/3	7/2	
AM630 (1 µM)	Tat(−)	8/3	8/2	31/10	8/3	7/2	30/10
	Tat(+)	7/3	8/2		8/3	7/2	
0 Ca^{2+}	Tat(−)	14/4	8/3	38/12	9/4	8/3	29/12
	Tat(+)	7/2	9/3		6/2	6/3	
$CdCl_2$ (1 µM)	Tat(−)	8/3	8/3	38/13	4/2	6/3	24/11
	Tat(+)	7/3	15/4		5/2	9/4	
Thapsigargin (200 µM)	Tat(−)	9/3	7/2	31/11	5/3	5/2	27/11
	Tat(+)	7/3	8/3		10/3	7/3	

* Sample size is the same for frequency and amplitude, [$]Control data are the data depicted in **Figure 2** of the main manuscript.

Table S2. Levels of eCB and non-eCB lipids in the prefrontal cortex of HIV Tat transgenic mice expressed in pg/mg as mean and SEM *.

Lipids	Genotype	Female			Male		
pg/mg		Mean	SEM	*n*	Mean	SEM	*n*
**2-AG**	Tat(−)	2462.8	311.62	9	2436.5	302.14	9
	Tat(+)	1927.8	143.69	8	2925.3	374.03	8
**2-LG**	Tat(−)	312.9	46.54	9	253.3	28.06	9
	Tat(+)	303.5	25.43	8	335.1	36.35	8
**AEA**[#,$]	Tat(−)	13.2	1.20	9	12.1	1.91	9
	Tat(+)	18.5	0.53	8	8.6	0.85	8
**DEA**[#,$]	Tat(−)	2.3	0.13	9	2.0	0.29	9
	Tat(+)	2.9	0.13	8	1.7	0.10	8
**DHEa**[#,$]	Tat(−)	19.7	1.83	9	16.7	2.54	9
	Tat(+)	26.2	1.01	8	13.8	0.97	8
**LEA**[#,$]	Tat(−)	11.6	1.19	9	8.3	1.44	9
	Tat(+)	15.5	1.09	8	7.1	0.69	8
**NAGly**[*,$]	Tat(−)	39.3	4.32	9	39.7	5.47	9
	Tat(+)	57.0	7.24	8	27.5	3.34	8
**OEA**[*]	Tat(−)	23.9	2.74	9	18.7	3.04	9
	Tat(+)	28.0	2.21	8	17.7	1.28	8
**PEA**[*]	Tat(−)	53.0	5.14	9	47.5	6.27	9
	Tat(+)	63.6	7.43	8	43.2	3.41	8
**POEA**[#,$]	Tat(−)	4.8	0.41	9	4.2	0.61	9
	Tat(+)	6.1	0.32	8	3.5	0.30	8
**SEA**[*]	Tat(−)	60.7	9.32	9	43.3	7.35	9
	Tat(+)	76.7	10.03	8	51.4	4.59	8

* Two-way ANOVAs were conducted with Sex and Genotype as between-subjects factors. [#]denotes Sex effect, $p < 0.05$; [$]denotes Sex x Genotype interaction, $p < 0.05$. See ANOVA results in **Table 1** of the main manuscript, *n*, sample size/group.

Supplemental Figures

Figures

Figure S1

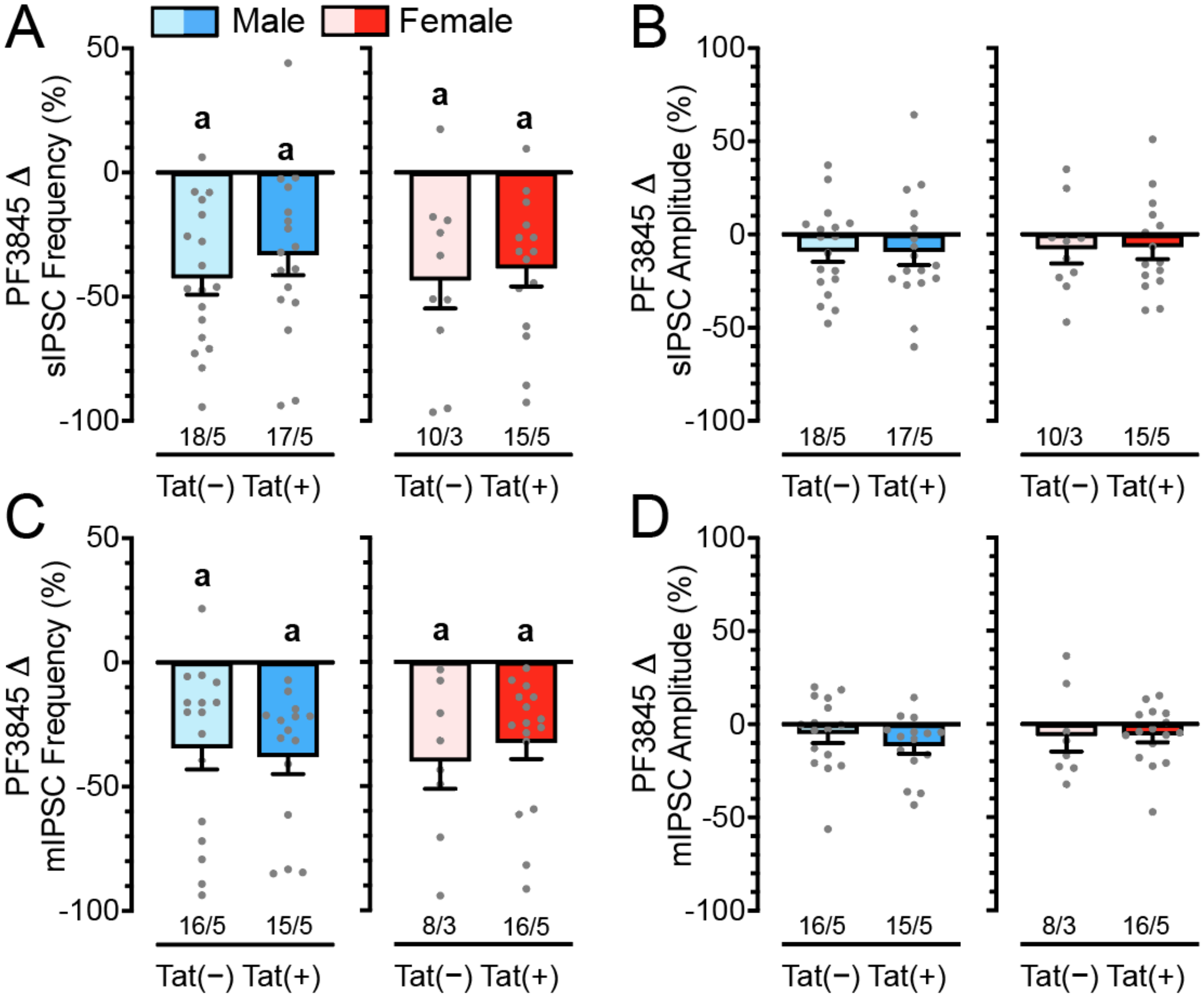

Figure S1. PF3845 significantly induces a decrease in the IPSC frequency (% change) in mPFC neurons independent of Tat induction and sex. (A) PF3845-induced change on sIPSC frequency was significant for all groups, with PF3845 significantly inhibiting percent sIPSC frequency similar across all groups. No effects were noted for sex and genotype. **(B)** For PF3845-induced change on sIPSCs amplitude no significant effect was noted, thus, PF3845 did not significantly inhibit percent sIPSC amplitude for any group. **(C)** PF3845-induced change on mIPSC frequency was significant for all groups, with PF3845 significantly inhibiting percent mIPSC frequency similar across all groups. No effects were noted for sex and genotype. **(D)** For PF3845-induced change on mIPSCs amplitude no significant effect was noted, thus, PF3845 did not significantly inhibit percent mIPSC amplitude for any group. Data are percent PF3845-induced change (Δ; mean ± SEM) separated by Sex and Genotype. Statistical significance was assessed by one-sample *t*-tests and ANOVA followed by Bonferroni's post hoc test when appropriate; [a]$p < 0.001$ vs. control (0%). In all panels, sample size is indicated as (cells/mice).

Figure S2

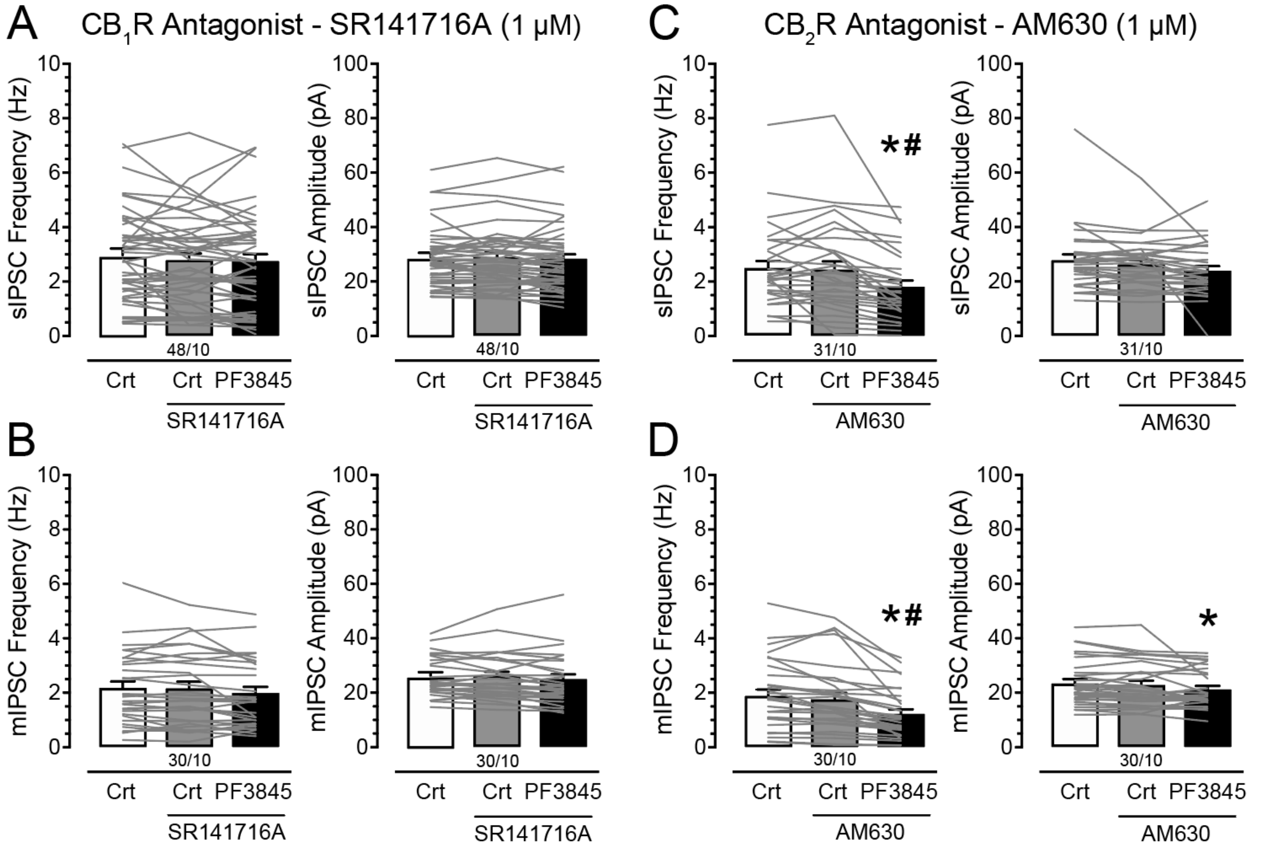

Figure S2. PF3845 effects on IPSCs in mPFC neurons are blocked by CB$_1$R antagonist SR141716A but not CB$_2$R antagonist AM630. No significant differences were noted for Sex and Genotype on any sIPSC measure and are therefore collapsed across. **(A-B)** Recordings of IPSCs for CB$_1$R antagonist treatment condition SR141716A (1 μM). **(A)** SR141716A alone and in combination with PF3845 (1 μM) showed no significant differences on sIPSC frequency and amplitude compared to control condition, indicating the CB$_1$R antagonist SR141716A was able to block the downregulating effects of PF3845 on sIPSCs. **(B)** Similarly, no significant effects were noted on the mIPSC frequency and amplitude. **(C-D)** Recordings of IPSCs for CB$_2$R antagonist treatment condition AM630 (1 μM). **(C)** AM630 alone showed no significant effects on sIPSC frequency or amplitude compared to control condition before AM630 treatment, whereas AM630 in combination with PF3845 (1 μM) significantly decreased sIPSC frequency compared to control or AM630 alone, indicating pretreatment of AM630 did not prevent the PF3845-induced decreases in the mean frequency of sIPSCs. No significant effects were noted on sIPSC amplitude. **(D)** Similarly, pretreatment of AM630 did not block the downregulating effects of PF3845 on mIPSCs and AM630 in combination with PF3845 significantly decreased the mean frequency and amplitude of sIPSCs. Data are raw data (mean ± SEM) collapsed across Sex and Genotype. Statistical significance was assessed by ANOVA followed by Bonferroni's post hoc test; $^{*}p < 0.05$ vs. control (before treatment); $^{\#}p < 0.05$ vs. corresponding counterpart before PF3845 treatment. In all panels, sample size is indicated as (cells/mice); please see **Supplemental Table S1** for specific information on sample size for sex and genotype. Crt, Control.

Figure S3

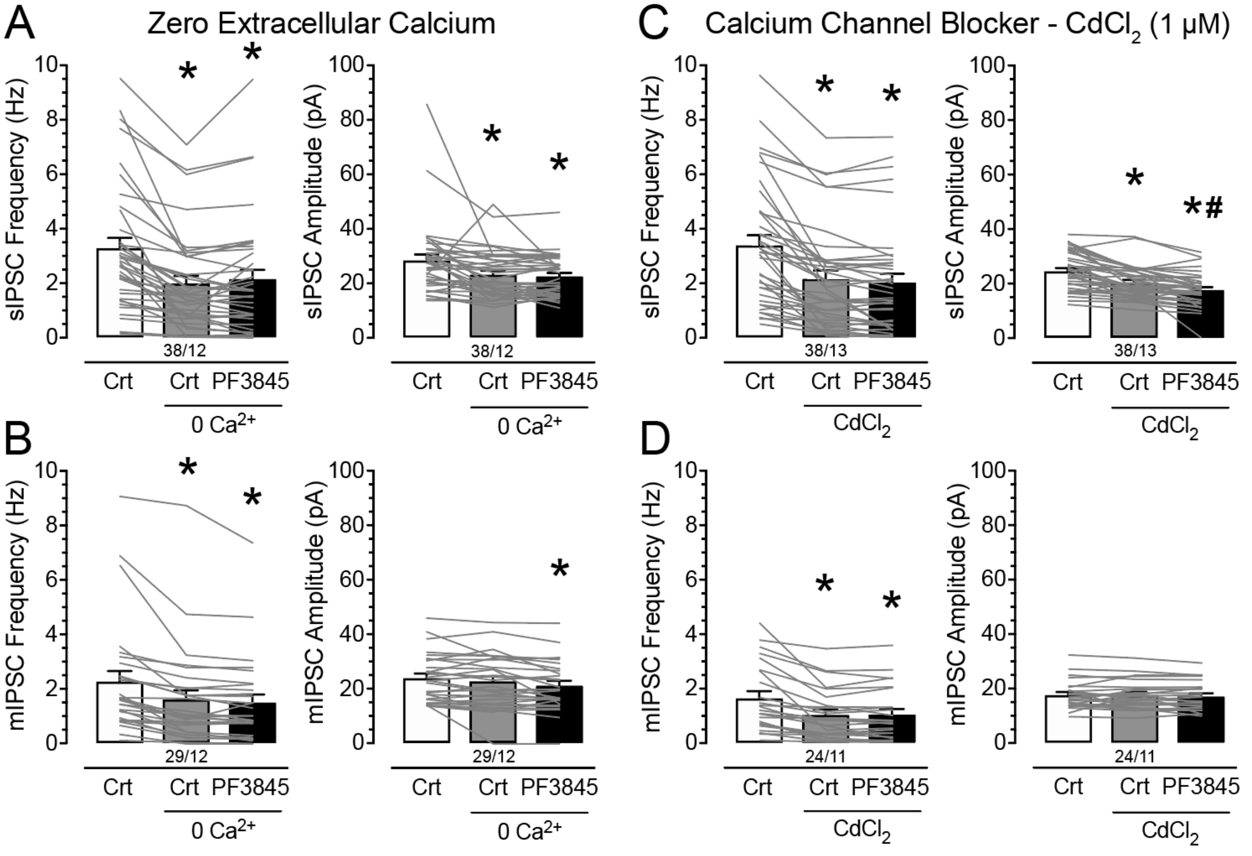

Figure S3. Effects of PF3845 on IPSCs in mPFC neurons are blocked in the absence of extracellular calcium. No significant differences were noted for Sex and Genotype on any sIPSC measure and are therefore collapsed across. **(A-B)** Recordings of IPSCs for zero extracellular calcium treatment condition. **(A)** Removing extracellular calcium from the aCSF in the presence or absence of PF3845 (1 μM) significantly downregulated sIPSC frequency or amplitude compared to control condition (before removal of 0 Ca^{2+}). Importantly, the 0 Ca^{2+} ± PF3845 condition was not significantly different from 0 Ca^{2+}, indicating PF3845 had no significant effect on sIPSC frequency or amplitude in the absence of extracellular calcium. **(B)** Similarly, mIPSC frequency or amplitude was significantly downregulated by 0 Ca^{2+} ± PF3845 (except for mIPSC amplitude at 0 Ca^{2+}) with PF3845 showing no further downregulation in the absence of extracellular calcium compared to the 0 Ca^{2+} condition. **(C-D)** Recordings of IPSCs for $CdCl_2$ (200 μM) treatment condition. **(C)** Application of $CdCl_2$ significantly downregulated sIPSC frequency or amplitude in the presence or absence of PF3845 (1 μM) compared to control condition (before $CdCl_2$ treatment). Interestingly, whereas PF3845 had no significant effect on sIPSC frequency in the presence of $CdCl_2$, PF3845's significant downregulating effects on the amplitude of sIPSCs were maintained ($CdCl_2$ vs. $CdCl_2$ + PF3845), indicating that other channels and receptors involved in Ca^{2+} influx contribute to PF3845-induced effects on GABAergic neurotransmission postsynaptically. **(D)** For mIPSCs, application of $CdCl_2$ significantly downregulated mIPSC frequency for $CdCl_2$ ± PF3845 conditions with PF3845 showing no further downregulation in the absence of extracellular calcium compared to the $CdCl_2$ condition. Data are raw data (mean ± SEM) collapsed across Sex and Genotype. Statistical significance was assessed by ANOVA followed by Bonferroni's post hoc test; $^*p < 0.05$ vs. control (before treatment); $^\#p < 0.05$ vs. $CdCl_2$ (200 μM). In all panels, sample size is indicated as (cells/mice); please see **Supplemental Table S1** for specific information on sample size for sex and genotype. Crt, Control.

Figure S4

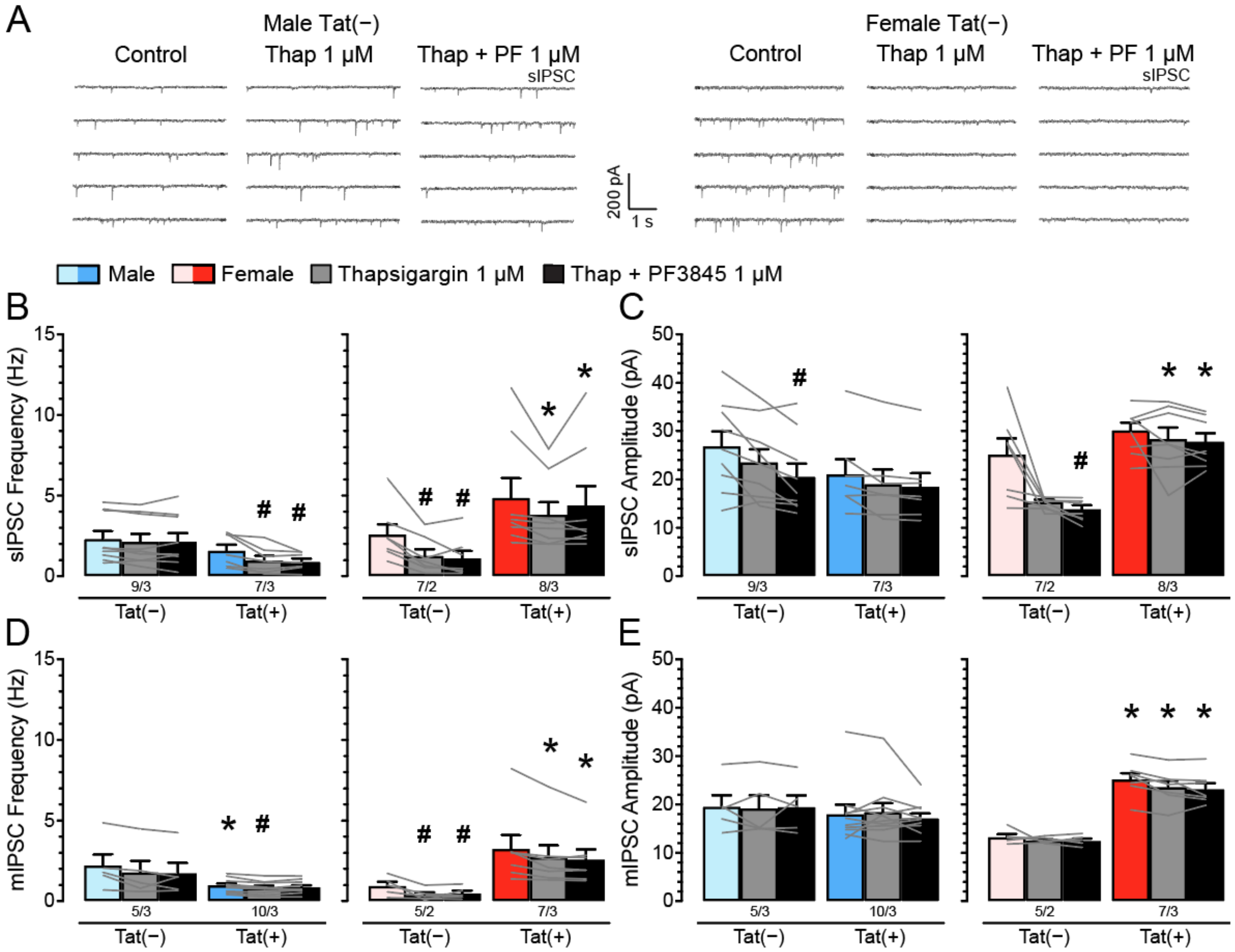

Figure S4. Depletion of intracellular calcium stores via thapsigargin affects IPSCs in mPFC neurons depending on sex and Tat induction and blocks PF3845's downregulating effects similarly across groups. (A) Representative traces of male and female Tat(−) mice show sIPSCs before and after thapsigargin (1 μM) ± PF3845 (1 μM) bath application. **(B)** Tat(+) females indicated higher sIPSCs amplitudes in the presence of thapsigargin compared to Tat(−) females. In Tat(+) males and Tat(−) females the application of thapsigargin (200 μM) significantly downregulated sIPSC frequency in the presence or absence of PF3845 (1 μM) but did not show an effect in the other two groups [Tat(−) males, Tat(+) females]. For all groups, PF3845 (1 μM) had no significant effect on sIPSC frequency in the presence of thapsigargin (200 μM), indicating that depletion of intracellular calcium stores with thapsigargin blocked PF3845's downregulating effects on sIPSC frequency similarly across groups. **(C)** For the mean amplitude of sIPSCs Tat(+) females indicated higher sIPSCs amplitudes in the presence of thapsigargin (200 μM) compared to Tat(−) females, similar to sIPSC frequencies. The application of thapsigargin (200 μM) significantly downregulated sIPSC amplitude in Tat(−) females and Tat(−) males [in the presence of PF3845], without any significant effects of PF3845 in any group. **(D)** For mIPSCs, Tat(+) females indicated higher mIPSCs frequencies in the presence of thapsigargin (200 μM) compared to Tat(−) females, whereas Tat(+) males indicated significant lower mIPSC frequencies compared to Tat(−) males in the control condition. Further Tat(+) males and Tat(−) females indicated a significant downregulation of mIPSC frequency with the application of thapsigargin (200 μM) in the presence and/or absence of PF3845 (1 μM) compared to the control conditions, whereas no effect was noted for Tat(+) males and Tat(−) females. For all groups, PF3845

(1 μM) had no significant effect on mIPSC frequency in the presence of thapsigargin (200 μM), indicating that depletion of intracellular calcium stores with thapsigargin blocked PF3845's downregulating effects on mIPSC frequency similarly across groups. **(E)** For the mean amplitude of sIPSCs Tat(+) females indicated higher sIPSCs amplitudes compared to Tat(−) females in all three conditions. The application of thapsigargin and/or PF3845 did not reveal any significant effects on sIPSC amplitude. Data are raw data (mean ± SEM) separated by Sex and Genotype. Statistical significance was assessed by ANOVA followed by Bonferroni's post hoc test or planned comparisons; *p < 0.05 vs. corresponding Tat(−) counterpart; #p < 0.05 vs. corresponding counterpart before treatment conditions. In all panels, sample size is indicated as (cells/mice). Thap, thapsigargin, PF, PF3845.
